# Proteomic changes upon treatment with semaglutide in individuals with obesity

**DOI:** 10.1038/s41591-024-03355-2

**Published:** 2025-01-03

**Authors:** Lasse Maretty, Dipender Gill, Lotte Simonsen, Keng Soh, Loukas Zagkos, Michael Galanakis, Jonas Sibbesen, Miquel Triana Iglesias, Anna Secher, Dirk Valkenborg, Jonathan Q. Purnell, Lotte Bjerre Knudsen, Abd A. Tahrani, Milan Geybels

**Affiliations:** 1 Data Science, Novo Nordisk A/S, Søborg, Denmark; 2 Department of Epidemiology and Biostatistics, School of Public Health, Imperial College London, London, UK; 3 Sequoia Genetics, London, UK; 4 Obesity Research, Novo Nordisk A/S, Måløv, Denmark; 5 Center for Statistics and Data Science Institute, Hasselt University, Hasselt, Belgium; 6 Brain and Adipose Biology, Novo Nordisk A/S, Måløv, Denmark; 7 Oregon Health & Science University (OHSU), Portland, OR USA; 8 Chief Scientific Advisor Office, Novo Nordisk A/S, Måløv, Denmark; 9 Medical & Science, Novo Nordisk A/S, Søborg, Denmark; 10 Department of Metabolism and Systems Science, University of Birmingham, Birmingham, UK; 11 Present Address: QIAGEN A/S, Aarhus, Denmark; 12 Present Address: Genmab A/S, Valby, Denmark

**Keywords:** Obesity, Proteomic analysis, Proteomics

## Abstract

Obesity and type 2 diabetes are prevalent chronic diseases effectively managed by semaglutide. Here we studied the effects of semaglutide on the circulating proteome using baseline and end-of-treatment serum samples from two phase 3 trials in participants with overweight or obesity, with or without diabetes: STEP 1 (*n* = 1,311) and STEP 2 (*n* = 645). We identified evidence supporting broad effects of semaglutide, implicating processes related to body weight regulation, glycemic control, lipid metabolism and inflammatory pathways. Several proteins were regulated with semaglutide, after accounting for changes in body weight and HbA_1c_ at end of trial, suggesting effects of semaglutide on the proteome beyond weight loss and glucose lowering. A comparison of semaglutide with real-world proteomic profiles revealed potential benefits on disease-specific proteomic signatures including the downregulation of specific proteins associated with cardiovascular disease risk, supporting its reported effects of lowering cardiovascular disease risk and potential drug repurposing opportunities. This study showcases the potential of proteomics data gathered from randomized trials for providing insights into disease mechanisms and drug repurposing opportunities. These data also highlight the unmet need for, and importance of, examining proteomic changes in response to weight loss pharmacotherapy in future trials.

## Abstract (web summary)

Using serum samples collected from participants of the STEP 1 and STEP 2 trials, the authors have uncovered changes to the proteome upon semaglutide treatment, which can shed light on the mechanism of action of the drug driving its benefits in obesity-related conditions.

## Main

Obesity prevalence continues to increase, with the global number of affected individuals expected to double from 988 million in 2020 to almost 2 billion in 2035^1^. This makes obesity one of the leading risk factors for multimorbidity^2^, negatively impacting metabolic, cardiovascular (CV), mental and physical health, as well as mortality^3,4^. Recent advances in achieving weight loss through lifestyle behavioral interventions, pharmacotherapy, metabolic bariatric surgery or a combination of these effectively reduce the economic and health impact of obesity and improve the quality of life of affected individuals^5^. With these successes, momentum is building to identify novel and more effective treatment strategies for obesity.

Proteomics is the large-scale study of the structure and function of proteins^6^ using high-throughput platforms. It has the potential to offer numerous insights, including improved understanding of disease pathophysiology, delineation of mechanisms of action for current treatment strategies, development of biomarkers to predict treatment response and disease progression, and identification of novel therapeutic targets^7^. The SomaScan® aptamer-based proteomic platform was the first high-throughput platform used in large-scale studies^8^ capable of simultaneously measuring the relative abundance of thousands of proteins from small sample volumes.

Weight loss following dietary interventions and bariatric surgery is associated with changes to the circulating proteome, including proteins related to inflammatory and metabolic pathways^9,10^. However, to the best of our knowledge, published studies reporting the impact of pharmacotherapy-mediated weight loss on the proteome are lacking.

Semaglutide is a long-acting glucagon-like peptide-1 (GLP-1) analog. In the United States, once-weekly subcutaneous semaglutide 1.0 mg was first approved in 2017 for use in adults with type 2 diabetes (T2D)^11^, followed by approval of an oral formulation in 2020^12^. Approval was based on the action of GLP-1 receptor agonists (GLP-1RAs) to lower glycated hemoglobin (HbA_1c_) levels by increasing glucose-dependent insulin release, reducing glucagon secretion and delaying gastric emptying^13,14^. However, GLP-1RAs also have separate effects on appetite regulation mediated by actions on central nervous system centers that result in weight loss^15,16^. Currently, once-weekly subcutaneous semaglutide 2.4 mg is approved for chronic weight management in adults with obesity, or overweight and with at least one weight-related comorbidity^17^, for reducing CV risk in adults with established cardiovascular disease (CVD) with obesity or overweight^18^, and in adolescents aged 12 years and older^19^, in conjunction with a reduced-calorie diet and increased physical activity plan.

In the randomized Semaglutide Treatment Effect in People with Obesity (STEP) 1 trial (intent to treat: 1,961 adults with overweight or obesity without T2D), once-weekly subcutaneous semaglutide 2.4 mg resulted in greater weight loss compared with placebo over a 68-week period (estimated treatment difference −12.4 percentage points, 95% confidence interval (CI) −13.4, −11.5; *P* < 0.001); half of the participants treated with semaglutide (50.5%) had a weight reduction of ≥15% compared with 4.9% assigned to placebo^20^. In the randomized STEP 2 trial (intent to treat: 1,210 adults with overweight or obesity and T2D), the estimated treatment difference from baseline to week 68 for semaglutide 2.4 mg versus placebo was −6.2 percentage points (95% CI −7.3, −5.2; *P* < 0.0001)^21^.

In our analysis, we used fasting serum samples collected at baseline and end of treatment in a large subset of participants from two randomized, double-blind, placebo-controlled phase 3 trials, STEP 1 and STEP 2, to investigate the effects of semaglutide treatment on the circulating proteome. By examining the effects following weight loss and also effects after accounting for weight loss on the proteome throughout the trial in those with and without T2D, and by comparing proteomic profiles with those of observational cohorts, we aimed to comprehensively study the proteomic effects of semaglutide treatment and elucidate the mechanism of action driving its benefits on weight- and obesity-related complications, and to determine its potential for use in new indications.

## Results

### Study participant characteristics

**Fig. 1:**
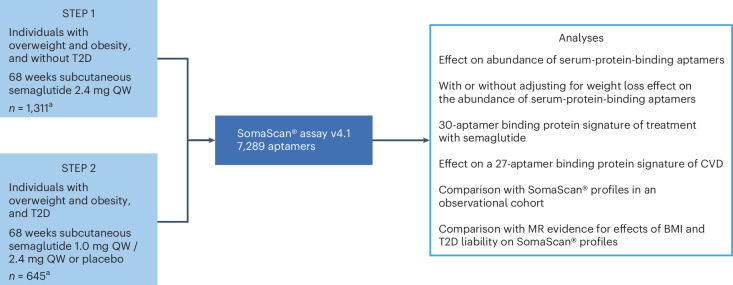
Study design. Of the 3,171 participants included in the STEP 1 and STEP 2 trials, 1,956 participants (STEP 1, *n* = 1,311; STEP 2, *n* = 645) consented to aptamer-based proteomic analyses using the SomaScan® assay v4.1. ^a^Participants included in the study who had an available biosample for proteomics profiling. The semaglutide 1.0 mg arm was excluded from most downstream analyses, except for the CVD risk analysis. MR, Mendelian randomization; QW, every week.

A total of 3,171 male and female participants were included in the STEP 1 and STEP 2 trials; please refer to the published papers for full details^20,21^. Of these, 1,956 participants (STEP 1, *n* = 1,311; STEP 2, *n* = 645) consented to aptamer-based proteomic analyses using SomaScan® assay v4.1 (SomaLogic) (Fig. 1). This assay uses 7,289 aptamers to measure the relative abundance of ~6,400 unique human proteins. After filtering for sample availability at both timepoints, individuals not on treatment at study end in both arms and vendor quality control, 1,728 participants (STEP 1, *n* = 1,133; STEP 2, *n* = 595) remained. For the majority of the analyses, only the placebo and semaglutide 2.4 mg arms from the STEP 2 trial were analyzed (*n* = 395). Baseline characteristics of consenting participants are shown in Table 1 and were similar to those of the overall study populations of the STEP 1 and STEP 2 trials^20,21^. Similarly, a post hoc analysis of changes in weight, waist circumference and HbA_1c_ for this subset of participants is shown in Supplementary Table 1.

**Table 1:** Characteristics of participants with fasting serum samples for aptamer-based proteomic analyses in STEP 1 and STEP 2

Characteristics	STEP 1, *n* = 1,311	STEP 2, *n* = 645
Age, mean ± s.d. (years)	47.5 ± 12.7	56.3 ± 10.8
Female sex, *n* (%)	955 (72.8)	321 (49.8)
Race or ethnic group, *n* (%)^a^		
White	984 (75.1)	388 (60.2)
Asian	162 (12.4)	183 (28.4)
Black or African American	65 (5.0)	55 (8.5)
Other	100 (7.6)	19 (2.9)
Hispanic or Latino ethnic group, *n* (%)^a^	135 (10.3)	77 (11.9)
Body weight, mean ± s.d. (kg)	106.0 ± 22.6	100.0 ± 21.5
BMI (kg m^−2^)		
Mean ± s.d. (kg m^−2^)	37.9 ± 6.7	35.7 ± 6.5
Distribution, *n* (%)		
<30	80 (6.1)	122 (18.9)
≥30 to <35	433 (33.0)	231 (35.8)
≥35 to <40	401 (30.6)	149 (23.1)
≥40	397 (30.3)	143 (22.2)
Waist circumference, mean ± s.d. (cm)	114.9 ± 14.9	114.4 ± 14.4
HbA_1c_, mean ± s.d. (%)	5.7 ± 0.3	8.1 ± 0.8
Prediabetes, *n* (%)^b^	570 (43.5)	0 (0)

^a^Race and ethnic group were reported by the investigator. The category of ‘other’ includes Native American, Hawaiian or other Pacific Islander, any other ethnic group and ‘not applicable’, the last of which is the way race or ethnic group was recorded in France.

^b^The presence of prediabetes was determined by investigators on the basis of available information (for example, medical records, concomitant medication and blood glucose variables) and in accordance with American Diabetes Association criteria^66^.

s.d., standard deviation.

### Effects of semaglutide on the circulating proteome

**Fig. 2:**
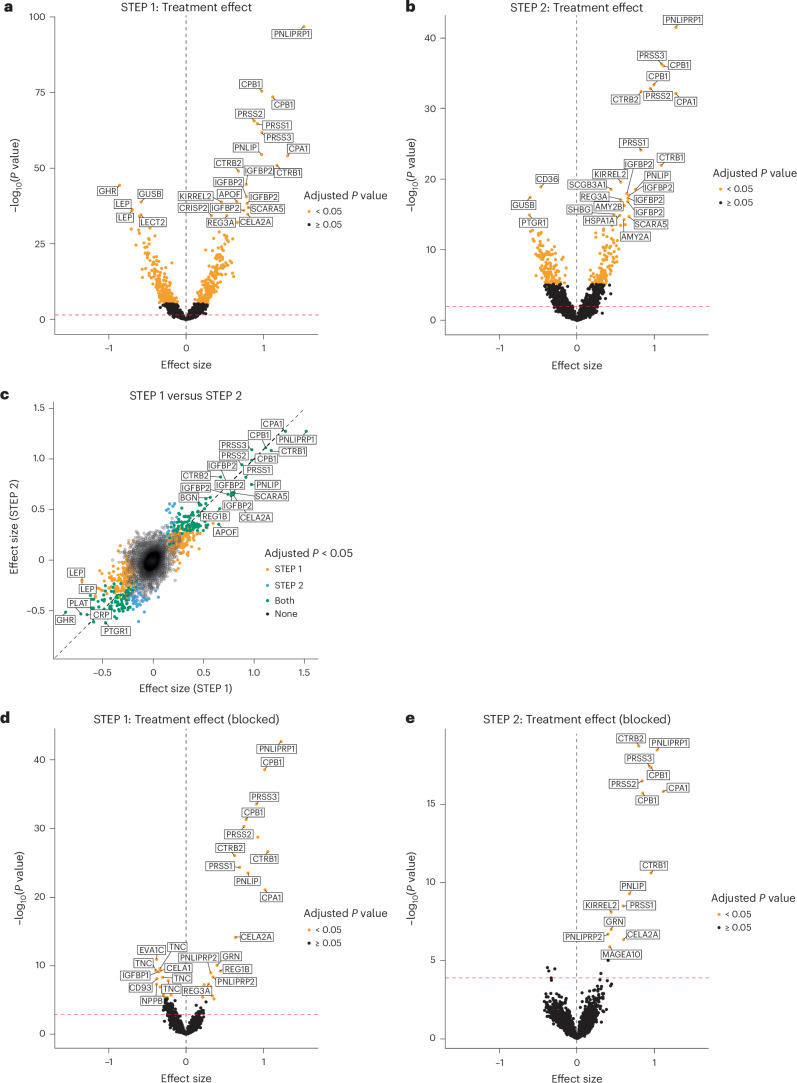
Effects of subcutaneous semaglutide versus effects of placebo on the circulating proteome. **a**,**b**, Effect sizes on protein levels in STEP 1 (**a**) and STEP 2 (**b**). **c**, Comparison between effect sizes in STEP 1 and STEP 2. **d**,**e**, Effect sizes on proteins that remained significant in the regression model after adjusting for both baseline and change in body weight and HbA_1c_ in STEP 1 (**d**) and STEP 2 (**e**). The dashed red line represents the FDR threshold. STEP 1: *n* = 1,133; STEP 2: *n* = 395. For **a**–**e**, effect sizes and *P* values were computed using linear regression. *P* values were corrected for multiplicity using Holm–Bonferroni correction. AMY2A and AMY2B, alpha-amylase 2A and 2B; APOF, apolipoprotein F; BGN, biglycan; CD36, cluster of differentiation 36; CELA1 and CELA2A, chymotrypsin-like elastase 1 and 2A; CPA1, carboxypeptidase A1; CPB1, carboxypeptidase B1; CRISP2, cysteine-rich secretory protein 2; CTRB1 and CTRB2, chymotrypsinogen B1 and B2; EVA1C, eva-1 homolog C; GUSB, glucuronidase beta; HSPA1A, heat shock protein family A member 1A; KIRREL2, kirre-like nephrin family adhesion molecule 2; LECT2, leukocyte cell-derived chemotaxin-2; LEP, leptin; NPPB, natriuretic peptide B; PLAT, plasminogen activator, tissue type; PNLIP, pancreatic lipase; PNLIPRP1 and PNLIPRP2, pancreatic lipase-related protein 1 and 2; PRSS1, PRSS2 and PRSS3, trypsin 1, 2 and 3; PTGR1, prostaglandin reductase-1; REG1B and REG3A, regenerating family member 1 beta and 3 alpha; SCARA5, scavenger receptor class A member 5; SCGB3A1, secretoglobin family 3A member 1; SHBG, sex-hormone-binding globulin.

**Extended Data Fig. 1:**
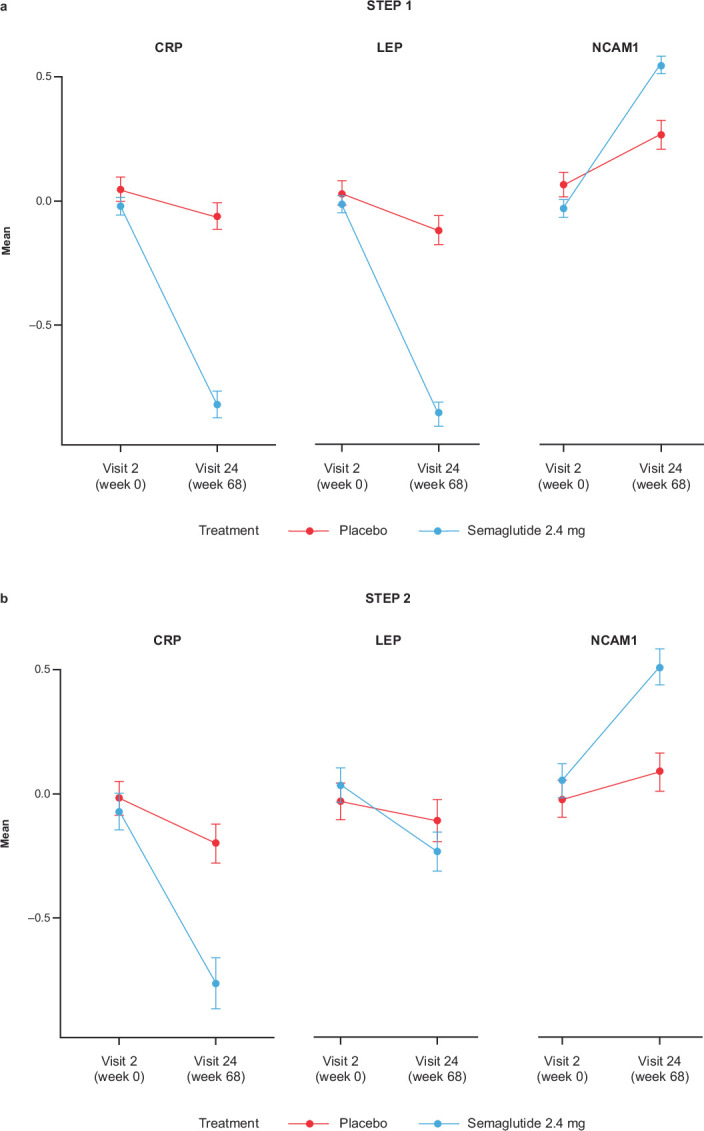
Effects of subcutaneous semaglutide versus placebo on the circulating proteome; relative abundance of specific proteins at baseline and week 68 in STEP 1 (a) and STEP 2 (b). Data are presented as mean values with error bars indicating 95% confidence intervals. STEP 1: n = 1,133; STEP 2: n = 395. CRP, C-reactive protein; LEP, leptin, NCAM1, neural cell adhesion molecule 1.

After 68 weeks of treatment, 495 protein targets (438 unique proteins) were identified to be significantly affected by semaglutide treatment (compared with placebo) in STEP 1 (*P* < 0.05, after Holm–Bonferroni correction for the 7,289 aptamers tested) (Fig. 2a and Supplementary Table 2), with 1,718 protein targets significant under false discovery rate (FDR) control. In the STEP 2 trial, the relative abundance of 277 protein targets (244 unique proteins) was changed significantly in response to semaglutide treatment relative to placebo (Fig. 2b and Supplementary Table 3; with 1,025 protein targets significant under FDR control). See Supplementary Tables 2 and 3 for FDR-adjusted *P* values (*q* values). Several of the proteins identified in STEP 1 and STEP 2 are known to be related to obesity and T2D pathophysiology and their associated complications, including C-reactive protein (CRP), adipokines (leptin, adiponectin), ghrelin, insulin-like growth factor binding protein (IGFBP), growth hormone receptor (GHR), neural cell adhesion molecule 1 (NCAM1) and netrin receptor (UNC5D), among others. Examples of the relative abundance of specific proteins at baseline and week 68 in STEP 1 and STEP 2 are shown in Extended Data Fig. 1. No effect of sex was detected in the proteomic response to semaglutide treatment in either STEP 1 or STEP 2 (Supplementary Tables 4 and 5).

We observed a high concordance between the proteomic response to treatment across STEP 1 and STEP 2 (Fig. 2c), but there were some differences between the two studies in terms of the impact of semaglutide on the measured proteome (Supplementary Tables 2, 3 and 6–8). A total of 33 proteins (including N-terminal pro B-type natriuretic peptide (NT-proBNP)) were significantly regulated in STEP 1 but not in STEP 2, although this may be owing to the larger sample size in STEP 1 than in STEP 2; this comparative analysis is considered exploratory. A list of the proteins that were regulated in STEP 2 but not in STEP 1 can be found in Supplementary Table 8.

**Extended Data Fig. 2:**
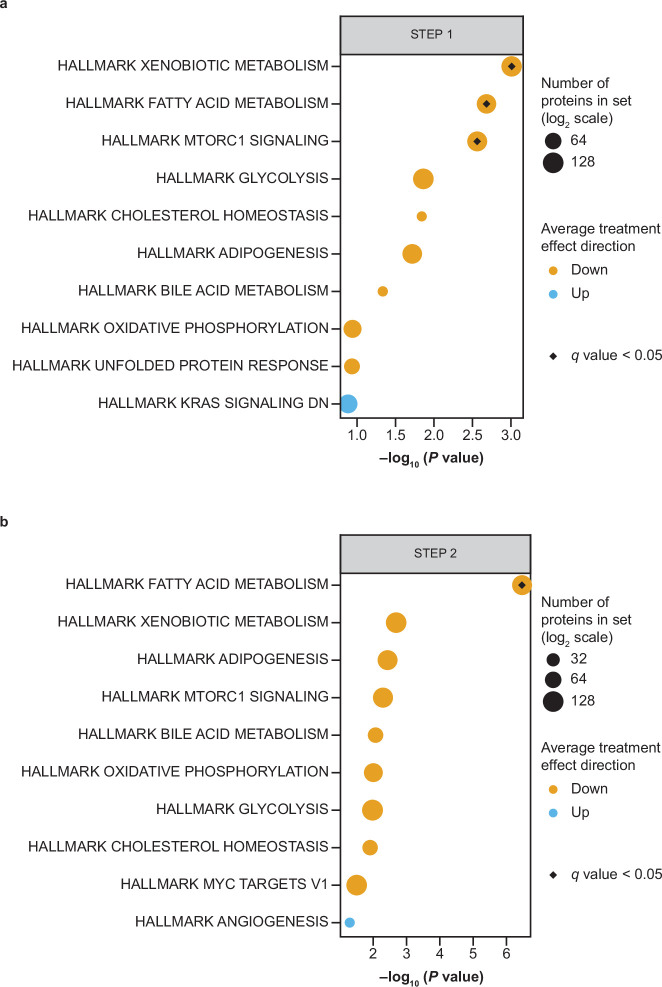
Pathways implicated by proteins affected by semaglutide treatment in STEP 1 (a) and STEP 2 (b). The figure shows the effect of semaglutide treatment (according to SomaScan®) on hallmark gene sets. Circle sizes visually indicate the number of proteins in a set (log_2_-scale). Black diamonds within the coloured circles indicate sets that are significantly affected by treatment (false discovery rate-adjusted *P* value [*q* value] < 0.05). Enrichment *P* values were computed using CameraPR. Results for the full list of gene sets are available in Supplementary Tables 9 and 10 for STEP 1 and STEP 2, respectively. KRAS, Kirsten rat sarcoma virus; MTORC1, mammalian target of rapamycin complex 1; MYC, myelocytomatosis oncogene.

**Extended Data Fig. 3:**
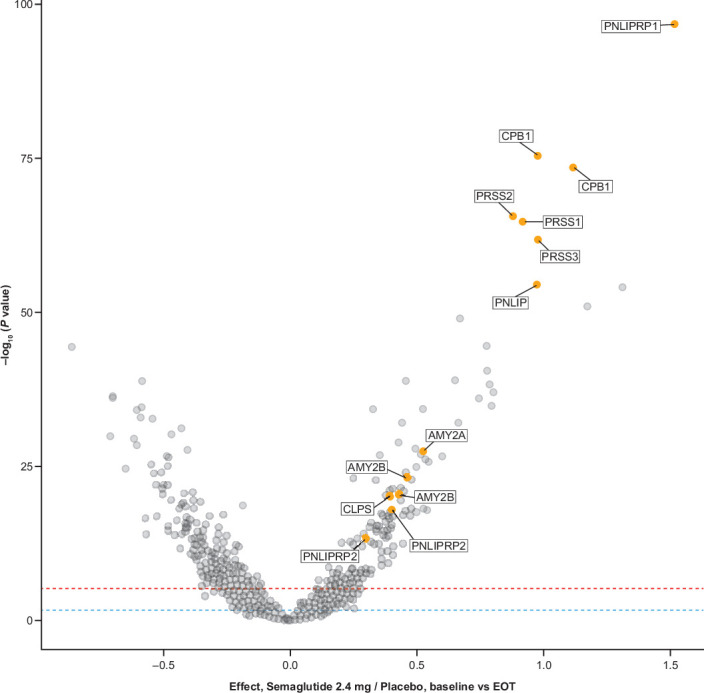
Proteins increased by semaglutide treatment are implicated in the exocrine pancreas. Effect size was normalized using the means and standard deviations at baseline to compare results across proteins, with positive and negative values indicating that semaglutide increased and decreased protein levels; −log_10_(*P* values) indicate the level of significance of the effect size (approximately 500 proteins [approximately 8–10% of the measured proteome] were significant). The red dashed line shows the Bonferroni correction limit, and the blue dashed line shows the false discovery rate. Effect sizes and *P* values were computed using linear regression. AMY2A/2B, alpha-amylase 2 A/2B; CLPS, caseinolytic protease subunit S; CPB1, carboxypeptidase B1; EOT, end of treatment; PNLIPRP1/2, pancreatic lipase-related protein 1/2; PNLIP, pancreatic lipase; PRSS1/2/3, trypsin 1/2/3.

Protein set analyses found that semaglutide treatment downregulated proteins involved in key biological pathways governing xenobiotic metabolism, fatty acid metabolism and mammalian target of rapamycin complex 1 (MTORC1) signaling, among others (Extended Data Fig. 2). In addition, levels of digestive enzymes secreted from the exocrine pancreas were increased by semaglutide (Extended Data Fig. 3).

### Weight-loss- and HbA_1c_-adjusted effects of semaglutide on the proteome

Adjusting for body weight and HbA_1c_ changes at the end of treatment identified 47 (38 unique proteins) and 15 (14 unique proteins) protein targets significantly altered by semaglutide treatment in STEP 1 and STEP 2, respectively (*P* < 0.05, after Holm–Bonferroni correction for the 7,289 aptamers tested (Fig. 2d,e), with 153 and 21 protein targets significant under FDR control, respectively) (Supplementary Tables 6 and 7). Significantly regulated proteins in this analysis are implicated in diverse biological effects (for example, cardiac stress, inflammation and lipid metabolism) and have previously been found to be increased in CVD or associated with higher CVD risk. Significantly downregulated proteins when adjusting for body weight effects in STEP 1 known to be associated with CVD risk included tenascin C (TNC), NT-proBNP, thrombospondin 2 (THBS2), complement component C1q receptor (cluster of differentiation 93 (CD93)), macrophage scavenger receptor 1—extracellular domain (MSR1) and angiopoietin-2 (ANGPT2). Other significantly downregulated proteins not related to CVD included secreted frizzled-related protein-4 (sFRP4) and liver fatty acid binding protein (LFABP), whereas granulin (GRN) was significantly upregulated. Similar to the previous analysis in which body weight and HbA_1c_ change were not included in the model, significantly upregulated proteins were enriched in digestive enzymes from the exocrine pancreas. Only one protein, melanoma-associated antigen 10A (MAGEA10), was significantly regulated by semaglutide in STEP 2, but not in STEP 1.

### Proteomic signature of semaglutide treatment

**Extended Data Fig. 4:**
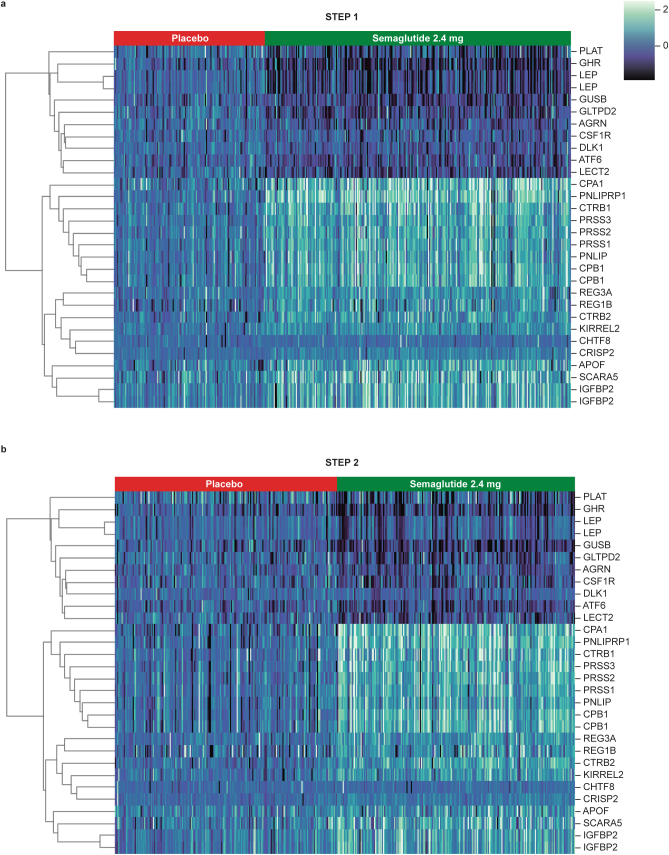
Thirty-aptamer signature of semaglutide treatment in STEP 1 (a) and STEP 2 (b). AGRN, agrin; APOF, apolipoprotein F; ATF6, activating transcription factor 6; CHTF8, chromosome transmission fidelity protein 8 homolog; CPA1, carboxypeptidase A1; CPB1, carboxypeptidase B1; CRISP2, cysteine-rich secretory protein 2; CSF1R, colony stimulating factor 1 receptor; CTRB1/2, chymotrypsinogen B1/2; DLK1, protein delta homolog 1; GHR, growth hormone receptor; GLTPD2, glycolipid transfer protein domain containing 2; GUSB, glucuronidase beta; IGFBP2, insulin-like growth factor-binding protein 2; KIRREL2, kirre-like nephrin family adhesion molecule 2; LECT2, leukocyte cell-derived chemotaxin-2; LEP, leptin; PLAT, plasminogen activator, tissue type; PNLIP, pancreatic lipase; PNLIPRP1, pancreatic lipase-related protein 1; PRSS1/2/3, trypsin 1/2/3; REG1B/3A, regenerating family member 1 beta/3 alpha; SCARA5, scavenger receptor class A member 5.

In STEP 1, statistical learning and feature selection were applied to derive a protein signature that distinguished participants receiving semaglutide from participants receiving placebo based on proteomic changes at end of treatment. The final trained signature included 30 aptamers (Extended Data Fig. 4a). The signature included proteins implicated in biological processes related to obesity and T2D (for example, adipogenesis, fatty acid metabolism, glycolysis and signaling pathways), consistent with the protein set analysis described above (Extended Data Fig. 2a), along with proteins related to fat mass and function, weight loss, CVD, endothelial function, lipid metabolism, pancreatic endocrine and exocrine function, inflammation and possibly cancer risk. The STEP 1 signature had a high classification performance (internal nested cross-validation area under the curve (AUC) = 0.94), showing that semaglutide treatment results in a specific serum proteomic signature. We then applied this signature in STEP 2, an external validation set, in which it showed similar performance for distinguishing semaglutide treatment from placebo (AUC = 0.93) (Extended Data Fig. 4b).

### Effect of semaglutide treatment on a proteomic signature of CVD risk

**Fig. 3:**
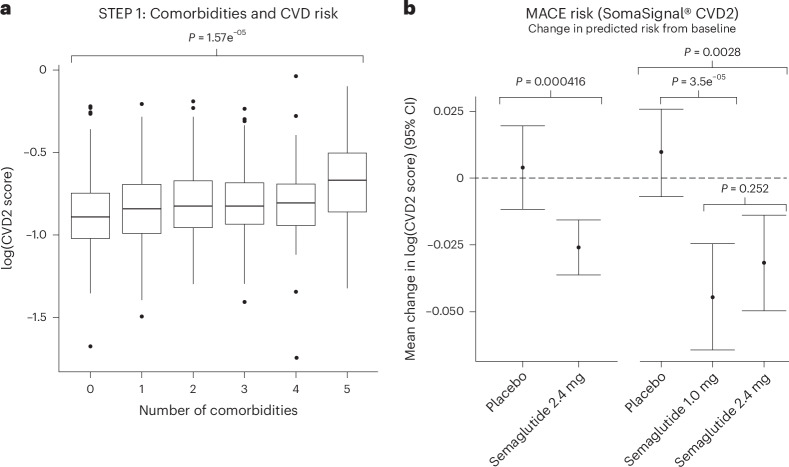
Relationship between number of comorbidities and predicted CVD risk in STEP 1 (a) and effect of semaglutide on predicted CVD risk in STEP 1 and STEP 2 (b). The CVD2 test predicts the risk of a new cardiovascular event within 4 years for patients who have already experienced a cardiovascular event^22^. **a**, log(CVD2 score) versus number of comorbidities: Kruskal–Wallis rank-sum test *P* = 1.57e^−05^. The center line and lower and upper bounds of the boxes represent the median and 1st quartile and 3rd quartile, respectively. The bottom and top whiskers indicate the minimum and maximum values, respectively, at 1.5× the inter-quartile range from the box bounds. STEP 1: *n* = 1,133; STEP 2: *n* = 395. **b**, Change from baseline in log(CVD2 score) across treatment groups: Wilcoxon rank-sum test *P* = 0.000416 (STEP 1, semaglutide 2.4 mg versus placebo), *P* = 3.5e^−05^ (STEP 2, semaglutide 1.0 mg versus placebo), *P* = 0.0028 (STEP 2, semaglutide 2.4 mg versus placebo) and *P* = 0.252 (STEP 2, semaglutide 2.4 mg versus semaglutide 1.0 mg). Data are presented as mean values with error bars indicating 95% CIs. Two-sided test was used. *P* values for pairwise arm comparisons in STEP 2 were corrected for multiple testing using the Holm–Bonferroni method. STEP 1: *n* = 1,133; STEP 2: *n* = 595. MACE, major adverse cardiovascular event.

A 27-protein score has previously been described that predicts secondary CVD risk over 4 years, based on large multicohort data and using the same SomaScan® technology used in our study^22^ (Extended Data Table 1). There was a trend for higher numbers of baseline comorbidities being associated with a higher CVD risk score, suggesting that the 27-protein score quantifies relative CVD risk in the STEP 1 cohort (Fig. 3a). Using data from STEP 1 and STEP 2, we found that semaglutide treatment reduced this CVD risk score compared with placebo (Fig. 3b). Similar statistically significant results were found when considering semaglutide doses of either 1.0 mg or 2.4 mg in STEP 2, with no additional risk reduction with the 2.4 mg dose versus the 1.0 mg dose.

### Comparison with Icelandic observational cohort data (deCODE)

**Fig. 4:**
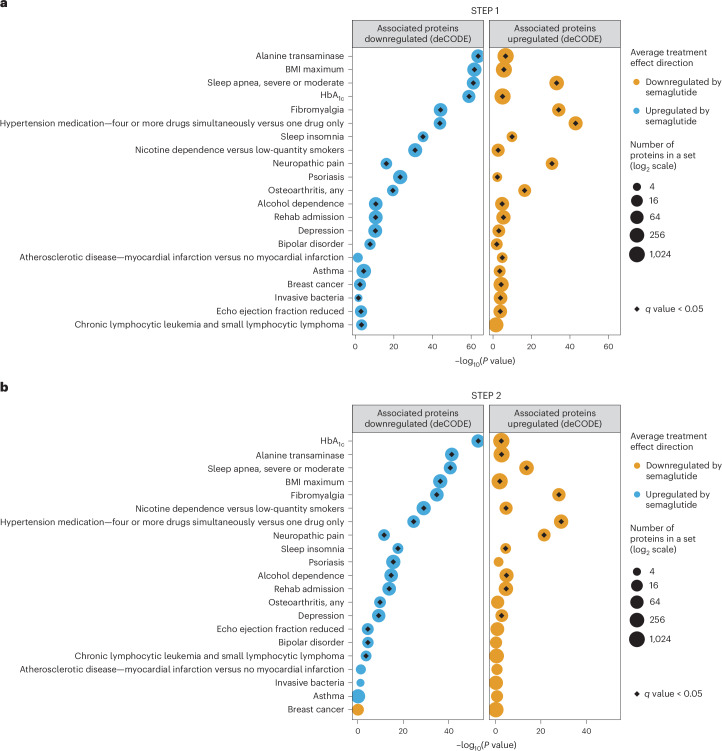
Effect of semaglutide in STEP 1 (a) and STEP 2 (b) on a selected set of proteomic signatures. **a**,**b**, Protein set analysis results for a selected set of proteomic signatures affected by semaglutide treatment (according to SomaScan®) in STEP 1 (**a**) and STEP 2 (**b**). STEP 1: *n* = 1,133; STEP 2: *n* = 395. Enrichment *P* values were computed using CameraPR. The protein sets (signatures) were created using data from a study by deCODE that estimated the associations between protein levels and clinical phenotypes in an observational cohort of 35,559 Icelanders^23^. For each phenotype, the significantly associated proteins were divided into those that were downregulated with the trait and those that were upregulated. Circle sizes visually indicate the number of proteins in a set (log_2_ scale). The black diamonds within the colored circles indicate sets that are significantly affected by treatment (FDR-adjusted *P* value (*q* value) < 0.05). For example, proteins downregulated with neuropathic pain in the deCODE study were upregulated by semaglutide, and proteins upregulated with neuropathic pain in the deCODE study were downregulated by semaglutide. Results for the full list of protein sets are available in Supplementary Tables 11 and 12 for STEP 1 and STEP 2, respectively.

We next compared the proteomic effect of semaglutide treatment in STEP 1 and STEP 2 with protein sets (signatures) associated with specific clinical phenotypes (Fig. 4). These sets were generated based on data from a previous study by deCODE (Methods), in which associations were estimated between protein levels and several clinical phenotypes in an observational cohort of 35,559 Icelanders^23^. For each phenotype, we divided the significantly associated proteins into those that were upregulated and those that were downregulated. Findings from this analysis indicated that the proteomic perturbations resulting from semaglutide might have a favorable impact on a wide range of metabolic parameters, obesity-related complications and other diseases (Fig. 4). For example, in STEP 1, semaglutide lowered proteins that are upregulated in fibromyalgia, hypertension, substance use disorders, neuropathic pain, osteoarthritis, psoriasis, depression, asthma, breast cancer and reduced ejection fraction, and increased proteins that are downregulated in these conditions (plus chronic lymphocytic leukemia and small lymphocytic lymphoma although *q* value ≥ 0.05) (Fig. 4a). Results for the full list of phenotypes for STEP 1 and STEP 2 are shown in Supplementary Tables 9 and 10 (hallmark analyses) and Supplementary Tables 11 and 12 (deCODE analyses), including *q* values.

### Comparison with Mendelian randomization analyses of body mass index and T2D genetic liability

**Fig. 5:**
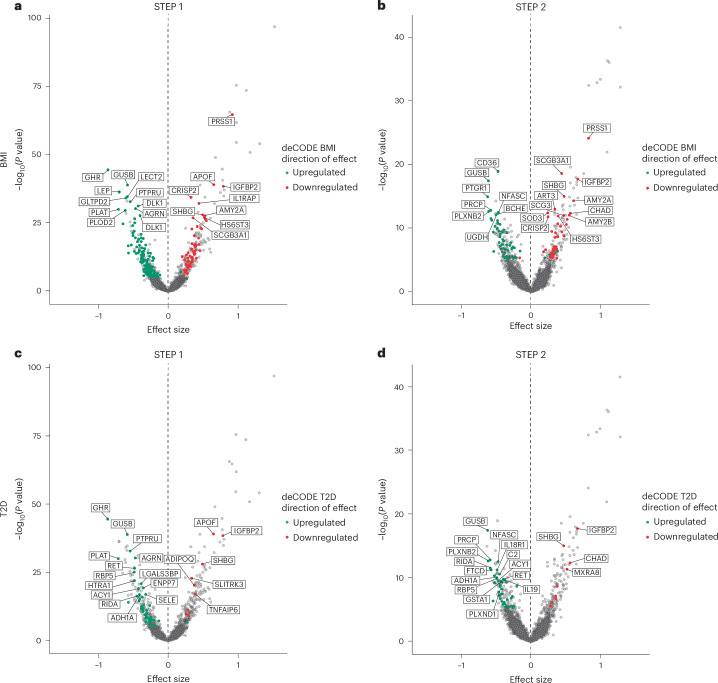
Comparison of the effects of semaglutide on the circulating proteome with effects associated with genetic liability to higher BMI and T2D. **a**,**b**, Proteins that were upregulated or downregulated by higher BMI in STEP 1 (**a**) and STEP 2 (**b**). **c**,**d**, Proteins that were upregulated or downregulated by T2D in STEP 1 (**c**) and STEP 2 (**d**). Negative values on the *x*-axis indicate proteins that have been downregulated by semaglutide, and positive values indicate proteins that have been upregulated by semaglutide. As such, the figure shows that semaglutide consistently reverses the protein expression patterns driven by higher BMI or T2D. STEP 1**:** *n* = 1,133; STEP 2**:** *n* = 395. Effect sizes and *P* values were computed using linear regression. *P* values were corrected for multiplicity using Holm–Bonferroni correction. ACY1, aminoacylase-1; ADH1A, alcohol dehydrogenase 1A; ADIPOQ, adiponectin; AGRN, agrin; ART3, ADP-ribosyltransferase 3; BCHE, butyrylcholinesterase; C2, complement component 2; CHAD, chondroadherin; DLK1, protein delta homolog 1; ENPP7, ectonucleotide pyrophosphatase/phosphodiesterase 7; FTCD, formimidoyltransferase cyclodeaminase; GLTPD2, glycolipid transfer protein domain containing 2; GSTA1, glutathione *S*-transferase A1; HS6ST3, heparan sulfate 6-*O*-sulfotransferase 3; HTRA1, high-temperature requirement A-1; IL1RAP, interleukin 1 receptor accessory protein; IL18RA, interleukin 18 receptor 1; IL19, interleukin 19; LGALS3BP, galectin 3 binding protein; MXRA8, matrix remodeling associated 8; NFASC, neurofascin; PLOD2, procollagen-lysine,2-oxoglutarate 5-dioxygenase 2; PLXNB2 and PLXND1; PRCP, prolylcarboxypeptidase; PTPRU, protein tyrosine phosphatase receptor type U; RBP5, retinol binding protein 5; RET, rearranged during transfection; RIDA, reactive intermediate imine deaminase A; SCG3, secretogranin III; SELE, selectin E; SLITRK3, slit guidance ligand and neurotrophic tyrosine receptor kinase-like family member 3; SOD3, superoxide dismutase 3; TNFAIP6, tumor necrosis alpha induced protein 6; UGDH, uridine phosphorylase-glucose 6-dehydrogenase.

Mendelian randomization analysis was performed to investigate the effect of genetic liability to lower body mass index (BMI) and lower T2D risk on the circulating proteome, using genetic association estimates for circulating proteins from the deCODE cohort. As expected, there was substantial overlap in the effects of subcutaneous semaglutide on serum protein levels with those associated with lower or higher genetic liability to BMI or T2D^23^ (Fig. 5). Specifically, proteins that were significantly elevated with higher genetic risk for increased BMI or T2D in the deCODE population were significantly downregulated by semaglutide and vice versa.

## Discussion

Using data from the STEP 1 and STEP 2 phase 3 trials, we found broad effects from subcutaneous semaglutide treatment in people with overweight or obesity (with and without T2D) on the circulating proteome compared with placebo. By comparing our data with real-world genetics and proteomics, we have shown that the abundance of many of the top-ranked proteins changed by semaglutide are implicated across a range of biological processes underlying BMI and T2D, from body weight regulation to glycemic control, to lipid metabolism, inflammatory pathways, immune function, adipose tissue function and several other obesity-related complications. A proteomic signature was identified that robustly predicted semaglutide treatment status. Moreover, our data suggest that semaglutide might lower CVD risk, even in populations without established CVD. In addition, although Mendelian randomization evidence generated using summary data from genome-wide association studies (GWAS) in this analysis shows that some effects of semaglutide treatment on the proteome may be mediated through body weight reduction and reduced T2D liability, analyses of the clinical trial data also support effects of semaglutide beyond those that can be explained by the observed weight loss and HbA_1c_ reductions.

Taken together, these clinical trial data of semaglutide pharmacotherapy for body weight reduction showcase the broad opportunities afforded by proteomic analyses, including offering insights into mechanisms of action and potential novel indications to be tested in clinical studies. Our findings also provide data supporting drug repurposing. Taking the impact of semaglutide on the whole proteome and comparing it with protein signatures identified with individual diseases from the deCODE analysis, we find that semaglutide might have a favorable impact on a variety of diseases and processes. For example, in both STEP 1 and STEP 2, semaglutide lowered proteins that are upregulated in fibromyalgia, hypertension, substance use disorders, neuropathic pain and depression, and increased proteins that are downregulated in these conditions. However, in patients without T2D (STEP 1), semaglutide also lowered proteins that are upregulated in osteoarthritis, psoriasis, asthma, breast cancer and reduced ejection fraction, and increased proteins that are downregulated in these conditions. A full comparison with deCODE is detailed in Supplementary Tables 11 and 12. As such, proteomic data from STEP 1 and STEP 2 provide insight into potential novel indications for semaglutide. However, such observations need to be considered as hypothesis generating, requiring testing in preclinical and clinical studies that confirm efficacy and ensure safety. For example, potential effects on alcohol dependency are supported by a recent real-world population propensity-score-matched study of 83,825 patients with obesity, which showed that semaglutide, compared with other non-GLP-1-based obesity medications, was associated with a 50% lower risk for incident and recurrent alcohol use disorders over a 12-month period regardless of T2D status^24^. These findings were replicated in a population of 598,803 patients with T2D^24^ and support the design of a clinical trial of semaglutide for the treatment of alcohol use disorder.

Not unexpectedly, the top upregulated proteins following semaglutide treatment in STEP 1 and 2 (including in the weight-adjusted analysis) were related to digestive enzymes and the exocrine pancreas. It is well established that multiple GLP-1RA treatments increase levels of blood pancreatic enzymes (amylase, lipase) as measured by laboratory assay, including in the STEP 1 trial^20^. Such increases rarely exceed the upper normal limit and are reversible after treatment discontinuation^25^. In the LEADER (Liraglutide Effect and Action in Diabetes: Evaluation of Cardiovascular Outcome Results) trial evaluating liraglutide (a GLP-1 analog), the observed mild elevations in pancreatic enzyme were not predictive of subsequent acute pancreatitis^26^. In addition, previous systematic reviews and meta-analyses have shown that GLP-1RA treatment in people with T2D was not associated with increased risk of pancreatitis^27,28^ or pancreatic cancer^28,29^.

While there was high concordance in the impact of semaglutide on the measured proteome between STEP 1 and 2, there were also some differences. Some proteins were regulated by semaglutide only in STEP 2 (Supplementary Table 8), while other proteins were regulated by semaglutide only in STEP 1. However, it is difficult to ascertain whether this was due to specific biology or the lack of statistical power after adjustment for multiplicity in STEP 2 due to a smaller sample size. Hence, we elected to list only the proteins that were regulated by semaglutide in STEP 2 but not STEP 1. Some of the differences might contribute to the variance in weight loss observed between patients without and with T2D (STEP 1 versus STEP 2). For example, metalloproteinase inhibitor 4 (TIMP4) was upregulated by semaglutide in STEP 2 but not STEP 1. Absence of TIMP4 ameliorated high-fat-diet-induced obesity in mice^30^. Polymorphisms in TIMP4 were also associated with weight loss responses to lifestyle behavioral interventions^31^. However, those differences need to be interpreted with caution considering the contrast in study populations and number, as well as the lower weight loss in STEP 2.

We used real-world omics from the deCODE study to gain a better understanding about the effects of semaglutide on other disease processes other than obesity. We used deCODE because it is the most contemporary and comprehensive resource of proteomic signatures of disease and health, and is a well-characterized cohort that has been extensively phenotyped and has contemporary proteomics data from the same platform used in our current study (albeit a previous version). Comparisons with proteomic profiles identified in the deCODE cohort (an observational cohort of 35,559 Icelanders^23^) allowed us to examine where semaglutide treatment might have benefits and assess its potential impact on a wide range of conditions, including CVD (atherosclerotic myocardial infarction and reduced ejection fraction on an echocardiogram); metabolic, inflammatory and mental health conditions; and cancer. Consistent with this, semaglutide treatment favorably reduced the cardiovascular disease 2 (CVD2) risk score calculated using a 27-protein proteomic model^22^. Although our analyses show that many of the proteomic perturbations resulting from semaglutide treatment are linked to changes in body weight and glycemia, the lack of a dose response on CVD risk with semaglutide 1.0 mg and 2.4 mg in STEP 2 supports semaglutide effects on CVD beyond weight loss. Furthermore, a prespecified analysis of the SELECT trial presented at the European Congress of Obesity 2024 revealed that the magnitude of this treatment effect with semaglutide was independent of the extent of achieved weight loss^32^. In our analyses, after adjusting for weight loss, approximately 90% of the initially statistically significant markers were no longer significant, suggesting that the remaining 10% of markers are not changed as a consequence of weight loss; however, additional investigation is warranted. We have provided a comprehensive list of changes in the measured proteome, which can be accessed and used in further analyses should comparable data become available.

Several GLP-1RAs, including semaglutide, have been shown to offer cardiovascular outcome benefit in people with T2D^33–35^. These benefits are now also evident in people with obesity at high CVD risk (or with known vascular disease) but without T2D, as shown by the favorable effects of semaglutide in the Semaglutide Effects on Heart Disease and Stroke in Patients with Overweight or Obesity (SELECT) clinical trial^36^. In our current analysis of the STEP 1 data, we found evidence that semaglutide might lower CVD risk even in populations not enriched for multiple CVD risk factors or with established CVD (primary prevention). Specific proteins reported to be upregulated in CVD (but downregulated with semaglutide treatment, after adjusting for effects of weight loss and HbA_1c_ reduction) include NPPB (NT-proBNP, released from cardiomyocytes on ventricular distension)^37^, ANGPT2 (involved in vascular remodeling and angiogenesis)^38^, CD93 (involved in cardiovascular homeostasis)^39^, MSR1 (involved in lipid metabolism and immune function)^40,41^, and THBS2 and TNC (both involved in extracellular matrix remodeling)^42^. Previous results indicate that TNC is downregulated by GLP-1RA treatment and correlates with incident CVD^43^, suggesting that TNC may act as a mediator, with downregulation contributing to the cardiovascular-protective effects of GLP-1RAs. This hypothesis will be tested using proteomic data from the SELECT trial^36^ for the six cardiovascular proteins listed above that were downregulated by semaglutide in STEP 1. Likewise, the favorable impact of semaglutide on inflammatory markers such as CRP and NT-proBNP levels might provide further insight into the potential benefit of semaglutide in patients with heart failure with preserved ejection fraction (HFpEF). This has been evaluated in the STEP-HFpEF (people with obesity and HFpEF without diabetes) and STEP-HFpEF DM (people with obesity and HFpEF with T2D) trials^44^. Indeed, data from STEP-HFpEF and STEP-HFpEF DM trials have shown a significant reduction in inflammatory markers such as CRP with semaglutide compared with placebo^45,46^. Despite the differences in study populations, our findings offer additional insight to support published studies that report favorable effects with semaglutide on CV outcomes.

The proteomic signature of semaglutide could possibly serve to assess intentional and nonintentional nonadherence with prescribed treatment, especially in those with modest weight loss^47^. It has been shown previously that 14% weight loss due to a very-low-energy diet (VLED) resulted in counter-regulatory changes in appetite-regulating hormones, including a significant increase in fasting levels of ghrelin and significant reductions in the fasting levels of leptin, GLP-1, peptide YY (PYY), cholecystokinin (CCK) and amylin^48^. During 1 year of follow-up after the end of the VLED intervention, the direction of these changes remained, but only the increase in ghrelin levels and reduction in fasting PYY levels were significant. Unlike the VLED, semaglutide did not appear to downregulate levels of satiety hormones such as PYY and amylin, and upregulated CCK levels. Semaglutide is known to improve satiety and reduce cravings for savory food, as well as improve the control of eating^49^, resulting in sustained weight loss over 104 weeks^50^. These actions are thought to be primarily mediated through central brain populations of GLP-1 receptors; for example, in the hypothalamus, semaglutide affects the pro-opiomelanocortin and cocaine- and amphetamine-regulated transcript (POMC/CART) and neuropeptide Y and Agouti-related protein (NPY/AGRP) neurons known to suppress appetite and stimulate food intake, respectively^51–53^. Such changes in appetite-regulating hormones might have contributed to the sustained weight loss and changes in the control of eating questionnaire responses observed in STEP 5 over a 2-year period^49,50^. The findings we report here suggest an additional impact of long-term semaglutide treatment in preventing some of the counter-regulatory (adaptive) changes in gut-hormone secretion conducive to weight regain following low-calorie dieting. These findings need to be interpreted with caution, as conditions were not optimized to capture meal-induced changes. Overall, many of the proteomic changes observed with semaglutide treatment overlap with findings following gastric bypass surgery (GBS)^54^. This is expected given that weight change has a marked effect on the circulating proteome and durable increases in postprandial GLP-1 levels are thought to favorably contribute to both weight and metabolic outcomes after GBS^55^. However, it is important to highlight that the GBS study used an older version of the SomaScan® assay that included a smaller number of proteomic markers (1,297), and that the comparison used 2 year post-GBS data versus the 1-year follow-up data in the STEP trials. We confirmed that four CV proteins (NPPB, CD93, MSR1 and TNC) were not changed following GBS, thereby suggesting that these effects are unique to semaglutide. In addition, some of the proteomic changes after GBS were not observed following semaglutide treatment. For example, contactin-4 (CNTN4) was upregulated after GBS but not semaglutide. CNTN4 has been described as a risk factor for alcohol use^56^, and increased alcohol use disorder has been widely reported after GBS^57^. On the other hand, real-world data suggest that semaglutide use in obesity is associated with reduction in alcohol use disorders^24^.

In addition to the effects beyond weight loss of semaglutide on proteins related to CVD (as discussed above), other significantly regulated proteins in this analysis have previously been implicated in other conditions such as Alzheimer’s disease (TNC^58^, granulin^59,60^), T2D (sFRP4 (refs. ^61,62^)) and metabolic-associated steatohepatitis (LFABP^63^).

This study has several strengths. We used fasting samples from well-characterized populations from two large phase 3 randomized controlled trials. With a high retention rate and inclusion of participants with and without T2D, we could explore the potential effects of semaglutide on reduction in CVD risk, either linked to or beyond weight loss and HbA_1c_, as well as potential novel indications that might help direct drug repurposing. Our study also has limitations. First, samples were collected only at baseline and the end of treatment for proteomic analyses. As a result, we did not have samples at early timepoints to compare the impact of semaglutide on the proteome during the weight loss and weight maintenance phases of treatment, nor in response to meal stimulation. Second, the methodology used for studying relative concentrations of protein-binding aptamers is semiquantitative; hence, we are not able to comment on absolute concentrations of proteins. Third, several of the differentially expressed proteins identified in our study have also been associated with BMI in a previously published observational study using the Olink affinity-based assay^64^, which supports our findings. In addition, while the NPPB SomaScan® aptamer has been shown to correlate with the NPPB immunoassay measurement^65^, we did not perform technical validation of individual markers. Therefore, we acknowledge the absence of paired data from the two different assays as a limitation of this study. To help overcome this limitation, our work focused on integrating data from multiple sources, including genetics, as well as complex signatures rather than individual proteins. For example, Mendelian randomization analysis using genetic instrumental variables was used to help validate some of the derived proteomic signatures for specific traits. Replication of the presented findings was also not performed. Lastly, a comparison of semaglutide-induced weight loss versus diet-induced proteomic changes would be highly valuable for this study; however, to our knowledge, such a dataset is not currently available.

In conclusion, we leveraged STEP 1 and STEP 2 clinical trial data to implicate semaglutide treatment in broad effects across the circulating proteome in people with obesity, with and without T2D. The observed effects highlight biological processes related to body weight regulation, glycemic control, lipid metabolism and inflammatory pathways. The proteomic perturbations observed with semaglutide treatment support favorable effects on a range of disease processes including CVD (as examined in the SELECT trial^36^). By triangulating with real-world evidence and Mendelian randomization analyses of GWAS summary data, the findings of this study collectively showcase the potential of randomized trial proteomic data for unraveling pharmacotherapeutic mechanisms of action and identifying novel indications.

## Methods

### Study participants, biosampling and proteomics profiling

A total of 3,171 men and women were randomized into the STEP 1 (NCT03548935; *n* = 1,961) and STEP 2 (NCT03552757; *n* = 1,210) trials. In the STEP 1 and STEP 2 trials, sex was self-reported. Participation in sampling for biobanking of serum and DNA was voluntary and not a prerequisite for participation in the trials. Biosamples were collected if a separate informed consent form had been signed, in accordance with local ethical and regulatory requirements, as described in the original studies^20,21^. The study protocol for the proteomic analyses was approved by the ethics committee for the Region of Southern Denmark (number H-21046833; a redacted protocol is provided separately).

Of 1,956 participants (STEP 1, *n* = 1,311; STEP 2, *n* = 645) who consented to donating biosamples, 1,728 participants (STEP 1, *n* = 1,133; STEP 2, *n* = 595) remained after filtering for sample availability at both timepoints, individuals not on treatment at study end in both arms and quality control flags (provided by SomaLogic). For the majority of the analyses, only the placebo and semaglutide 2.4 mg arms from the STEP 2 trial were analyzed (*n* = 395).

The SomaScan® assay v4.1 (SomaLogic) was used for profiling ~6,400 unique human proteins encompassing a diverse set of biological processes (for example, cancer, inflammation and cardiovascular function) and secreted, intracellular and extracellular proteins and domains (for example, receptors, kinases, growth factors and hormones)^67^. The SomaScan® assay v4.1 has been validated in human ethylenediaminetetraacetic acid plasma and serum^8^. SomaScan® uses chemically modified nucleotide sequences (aptamers) to transform a protein signal into a nucleotide signal that can be quantified using relative fluorescence on microarrays^8^.

### Data preprocessing

SomaLogic’s normalization procedure, including the adaptive normalization by maximum likelihood step, was used for the SomaScan® data set for all analyses as recommended by SomaLogic. Data were processed in R v4.3.1 (https://cran.r-project.org/bin/windows/base/old/4.3.1/) using the SomaDataIO package (v6.0.0) for loading raw proteomics data from .adat files and tidyverse packages (v2.0.0) for data processing. After filtering on samples passing SomaLogic quality control (RowCheck = =TRUE) and selecting aptamers targeting human proteins, the remaining data covering 7,289 aptamers were log_10_ transformed and, for each aptamer, all measurements standardized using the mean and standard deviation (s.d.) of samples obtained at baseline.

### Statistical analyses

#### Estimating effects of semaglutide on the circulating proteome

Linear regression was used to test for effects of treatment with semaglutide 2.4 mg relative to placebo on the change from baseline in relative protein concentration at week 68. For this analysis and all other analyses comparing protein abundance at week 68 to baseline, we excluded individuals in both the placebo and treatment arms that were not on treatment at week 68. The baseline level of the protein was included in the regression model in addition to the treatment indicator. Results were adjusted for multiple testing across aptamers using the Holm–Bonferroni procedure (0.05 level). All analyses were performed separately for STEP 1 and STEP 2. We chose to base the main analysis on the Holm–Bonferroni correction, as this is more conservative than the FDR.

A follow-up analysis was performed in which baseline and percentage change from baseline to week 68 (end of treatment) for both body weight and HbA_1c_ were included as additional covariates in the model.

We also evaluated the impact of semaglutide on the measured proteome in a single model for men versus women, including an interaction term between treatment and sex.

#### Proteomic signature of semaglutide treatment

Statistical learning was used to generate a proteomic signature of semaglutide treatment. Protein change at week 68 (versus baseline) for 7,289 protein-binding aptamers was used as the input for the algorithm. In addition to the above-mentioned preprocessing steps, the relative protein abundances were also 5% winsorized for this analysis. Feature selection was performed using the minimum redundancy maximum relevance algorithm^68^ and the logistic regression procedure, and fivefold cross-validation was used to derive optimal tuning parameters. The model was first generated using STEP 1 data, with internal cross-validation AUC evaluated as a metric of model precession. The final model was used for STEP 2 data to classify patients treated with semaglutide 2.4 mg from those treated with placebo, in which the AUC criterion was used.

#### Effect of semaglutide treatment on a proteomic signature of secondary CVD risk

Previous work generated and validated a 27-protein signature predicting a 4-year likelihood of myocardial infarction, stroke, heart failure or death in patients with established CVD^22^. The predicted event probabilities for each individual in STEP 1 and STEP 2 were provided by SomaLogic. The Wilcoxon rank-sum test was used to assess statistical significance on the change in log risk from baseline between semaglutide and placebo groups.

#### Comparison with observational cohort data

The deCODE study measured 4,907 protein targets in 35,559 Icelanders with the SomaScan® v4.0 multiplex aptamer assay and examined the association between plasma protein levels and 373 clinical phenotypes after adjusting for age, sex and multiplicity^23^. Comparisons with proteomic profiles identified in the deCODE cohort allowed us to examine where semaglutide treatment might have benefits and assess its potential impact on a wide range of conditions.

A total of 257,490 significant associations between SomaScan® biomarkers and any of the 373 phenotypes were identified in the deCODE study, after Bonferroni correction (0.05 significance level)^23^. Using the significant findings in deCODE, two sets of proteins were generated for each phenotype: (1) proteins positively associated with the trait and (2) proteins negatively associated with the trait. For each of the sets, we then compared the proteins in the set with the semaglutide treatment effect results from STEP 1 and STEP 2. More specifically, we first ranked all aptamers according to their test statistic estimate from the linear regression treatment-effect analysis (change in log protein at week 68 versus baseline). Next, for each deCODE protein set, we mapped the proteins in the set to all the ranked proteins from the previous step. For proteins that had multiple aptamers, the mean of the test statistic estimate across aptamers was used. Finally, for each set, we tested whether the proteins in the set were highly ranked in terms of treatment effect relative to proteins not in the set. This was performed using cameraPR (a ‘pre-ranked’ version of the competitive gene set method camera), which is part of the Bioconductor limma package^69,70^. CameraPR was run with default parameters using limma v3.52.4. Results were adjusted for multiple testing across protein sets using the default procedure in cameraPR (Benjamini and Hochberg FDR). The analysis was performed independently for STEP 1 and STEP 2. The same method (cameraPR) was used for the protein set analysis using the hallmark gene set collection^71^.

#### Comparison with Mendelian randomization analyses of BMI and T2D genetic liability

Genetic association data for BMI were obtained from a meta-analysis of GWAS conducted in 694,649 individuals^72^ and for T2D from the DIAMANTE consortium (80,154 cases and 853,816 controls)^73^. Genetic summary statistics for 4,907 aptamer-based protein measures were obtained from a GWAS conducted in 35,559 Icelanders^23^. Protein levels were measured using the SomaLogic SomaScan® v4.0 platform. All individuals in the analysis were of European ancestry. We selected single-nucleotide polymorphisms associated with BMI and T2D liability at the genome-wide significance level (*P* < 5 × 10^−8^) as genetic proxies. Single-nucleotide polymorphisms were clumped at a pairwise linkage disequilibrium *r*^2^ < 0.01 and a window of 1 Mb, using the 1000G European reference panel phase 3 (ref. ^74^). We explored the effect of BMI and T2D genetic liability on 4,907 plasma proteins using the random-effects inverse-variance-weighted Mendelian randomization method^75^. Associations that survived a Bonferroni procedure correction (0.05 level) were considered statistically significant. Mendelian randomization estimates (betas) were reported as an s.d. change in plasma proteins per one s.d. increase in genetically predicted BMI or per one log-odds increase in genetically predicted T2D liability.

### Reporting summary

Further information on research design is available in the Nature Portfolio Reporting Summary linked to this article.

## Online content

Any methods, additional references, Nature Portfolio reporting summaries, source data, extended data, supplementary information, acknowledgements, peer review information; details of author contributions and competing interests; and statements of data and code availability are available at 10.1038/s41591-024-03355-2.

## Supplementary information

Supplementary Information Legends of Supplementary Tables 1–12.

Reporting Summary

Supplementary Data Supplementary Tables 1–12.

## Figures and Tables

**Extended Data Table 1:**
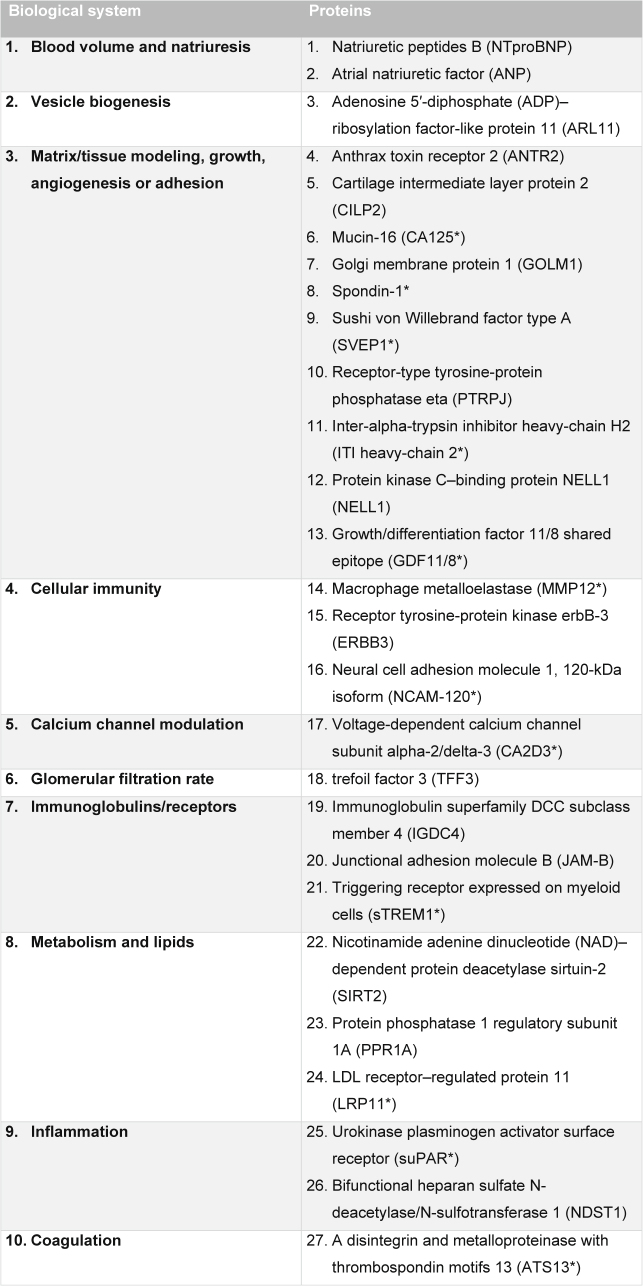
CVD2 test proteins

A 27-protein score has previously been described that predicts secondary CVD risk over 4 years, utilizing large multicohort data and the same SomaScan® technology used in our study^22^. This score was derived from plasma samples of 22,849 participants across nine clinical studies. The identified proteins were related to 10 biological systems, as summarized above.

## Data Availability

Proteomic results and summary association data are available through a dashboard at https://step-proteomics.azurewebsites.net/. Individual participant data from the STEP 1 and STEP 2 clinical trials can be shared in datasets in a de-identified and anonymized format. Access request proposals can be found at https://www.novonordisk-trials.com/. Data must not be used for commercial purposes. Data from the deCODE study are available in ref. ^23^. Data used in the gene set enrichment analysis are available in Supplementary Table 11 (ref. ^23^). GWAS summary statistics for aptamers are available at https://www.decode.com/summarydata/. Details regarding the hallmark gene set collection are provided in ref. ^71^. Data are available at https://www.gsea-msigdb.org/gsea/msigdb/.

## Code availability

Code for preprocessing SomaScan® proteomics data, performing statistical analyses and generating figures is available via Zenodo at 10.5281/zenodo.13356055 (ref. ^76^). R v4.3.1 is available at https://cran.r-project.org/bin/windows/base/old/4.3.1/.
