# Supplementary material for: Proteomic changes upon treatment with semaglutide in individuals with obesity
Source: Nat Med. 2025 Jan 3;31(1):267–77. doi: 10.1038/s41591-024-03355-2 (PMC11750704; doi:10.1038/s41591-024-03355-2)
Supplement: Supplementary file 1 — Legends of Supplementary Tables 1–12. [file 41591_2024_3355_MOESM1_ESM.pdf]

nature medicine

Supplementary information https://doi.org/10.1038/s41591-024-03355-2

Proteomic changes upon treatment with semaglutide in individuals with obesity

In the format provided by the authors and unedited

Supplementary Table 1 | Changes in weight, waist circumference and HbA_{1c} in patients who provided samples for the proteomics analysis in STEP 1 and STEP 2

Supplementary Table 2 | Proteins significantly changed by semaglutide treatment in STEP 1

Supplementary Table 3 | Proteins significantly changed by semaglutide treatment in STEP 2

Supplementary Table 4 | Proteins significantly changed by semaglutide treatment after adjusting for sex in STEP 1

Supplementary Table 5 | Proteins significantly changed by semaglutide treatment after adjusting for sex in STEP 2

Supplementary Table 6 | Proteins significantly changed by semaglutide treatment after adjusting for weight loss and HbA_{1c} reduction in STEP 1

Supplementary Table 7 | Proteins significantly changed by semaglutide treatment after adjusting for weight loss and HbA_{1c} reduction in STEP 2

Supplementary Table 8 | Proteins significantly changed by semaglutide treatment in STEP 2 but not in STEP 1

Supplementary Table 9 | Protein set analysis results in STEP 1 using Hallmark gene sets[1]

Supplementary Table 10 | Protein set analysis results in STEP 2 using Hallmark gene sets[1]

Supplementary Table 11 | Protein set analysis results in STEP 1 using gene sets created from observational cohort data (deCODE)[2]

Supplementary Table 12 | Protein set analysis results in STEP 2 using gene sets created from observational cohort data (deCODE)[2]

References

1. Liberzon, A. et al. The Molecular Signatures Database (MSigDB) hallmark gene set collection. *Cell Syst.* **1**, 417–425 (2015).
2. Ferkingstad, E. et al. Large-scale integration of the plasma proteome with genetics and disease. *Nat. Genet.* **53**, 1712–1721 (2021).

Protocol (ethical application for research in biological samples with dispensation from consent)

Title: Identification of biomarkers in patients with overweight or obesity

To:

De Videnskabsetiske Komitéer,
Region Hovedstaden
Regionsgården
Kongens Vænge 2
3400 Hillerød

Research Investigator/notifier:

[redacted]
Novo Nordisk
Vandtårnsvej 108-110
DK-2860 Søborg
Email: [redacted]
Phone: [redacted]

Sponsor:

Novo Nordisk A/S
Novo Allé 1
2880 Bagsværd
CVR number: 24256790
SE number: 62565314

Redacted protocol
includes redaction of personal identifiable information only.

.

Background

The prevalence of obesity has reached epidemic proportions and continues to increase[1]. Obesity is currently considered one of the most significant public health challenges worldwide due to its substantial medical, societal, and economic impact[2,3]. Obesity is associated with several health-related complications. Most concerning, obesity increases the risk of developing type 2 diabetes, cardiovascular disease and certain types of cancers, which are some of the leading causes of early death in these patients.[4]

Lifestyle intervention in the form of diet and exercise is first line treatment for obesity, but most people with obesity struggle to achieve and maintain their weight loss with diet and exercise alone.[5,6] During weight loss, the restricted food and energy intake is counteracted by adaptive biological responses to loss of weight[7-9]. The fall in energy expenditure and increase in appetite observed after a weight loss are associated with changes in a range of hormones[10]. Some of these changes result in altered physiology that promotes weight regain. Pharmacotherapy may therefore serve as a valuable adjunct to lifestyle intervention for individuals with obesity to achieve and sustain a clinically relevant weight loss, to improve comorbid conditions and to facilitate a healthier lifestyle.

Semaglutide is a glucagon-like peptide-1 (GLP-1) analogue and has a unique therapeutic potential for weight management, including weight loss and weight maintenance, due to its combined effects not only on body weight but also on glucose metabolism and other weight-related comorbidities[11]. Semaglutide 2.4 mg once weekly has been investigated in the STEP phase 3a trials[12,13] to support a weight management indication and has recently been approved for this use in the US and is currently under regulatory review for weight management in the EU and other countries. Semaglutide s.c. in doses of 0.5 mg and 1.0 mg once weekly is approved in several countries for treatment of adults with T2D under the tradename Ozempic®.

Rationale and hypothesis for exploring novel biomarkers in obesity

Overweight and obesity is traditionally classified based on the body mass index (BMI) with overweight classified as BMI of 25.0 to <30 kg/m^2 and obesity a BMI ≥30 kg/m^2; however, BMI is known to be an imperfect measure of excessive or abnormal body fat accumulation and has several limitations in predicting who will progress in their disease to develop obesity-related complications[14]. Investigations into the underlying biological processes of the association between adiposity, metabolic disorientation and complications have suggested several biomarkers as potential mediators. Heterogeneity has also been observed in the response to pharmacotherapy (including semaglutide) with no clear predictors based on the standard demographics features of the population.

Exploring novel prognostic and predictive biomarkers beyond BMI within obesity is therefore warranted to better understand prognosis and to characterise the obesity phenotype more precisely. Obesity biomarkers can include genomic, epigenomic, proteomic, and metabolomic markers whereof the two latter have recently been brought forward by newer high-throughput technologies. If successful in discovering new obesity biomarkers these could help physicians in selecting the patients that are most likely to progress in their disease and who would respond best to treatment.

Human biosamples (serum and DNA) collected for future research were collected in the completed STEP 1 and 2 clinical trials (trial IDs: NN9536-4373 and NN9536-4374) to facilitate future analysis to improve the understanding of progression of disease and the effects of semaglutide treatment. These biosamples have been used to establish a research biobank, owned and controlled by Novo Nordisk A/S and located at an external facility ([redacted]). The present protocol is part of a dispensation from consent application regarding use of the samples obtained for future research from trials NN9536-4373 and NN9536-4374.

The objectives of trials NN9536-4373 and NN9536-4374 were to investigate the effects of semaglutide s.c. 2.4 mg once-weekly on body weight loss, CV risk markers, glucose metabolism and other endpoints compared with placebo in subjects with overweight or obesity without type 2 diabetes (NN9536-4373) or with type 2 diabetes (NN9536-4374). For full protocols see appendix 1.

Subjects in the trials have signed a separate informed consent agreeing to store and use of blood samples for future research. This protocol will outline the concrete additional analyses to be done on some of the collected serum samples.

Aims

To measure circulating, epigenetic and genetic biomarkers from the completed trials NN9536-4373 and NN9536-4374 to investigate:

- if genetic predisposition, DNA methylation and baseline metabolite and protein profiles can predict development of obesity-related complications or treatment response in patients with overweight or obesity with and without T2D.

- the effect of semaglutide treatment versus placebo on protein profiles, biological pathways, or metabolites assessed at baseline and end of treatment to improve the understanding of its mode of action in the study population.

- the effect of semaglutide treatment versus placebo on DNA methylation and epigenetic ageing over time and how this potentially translates into measurable changes in the proteome and metabolome.

- disease biology, new drug targets and prevention strategies for people living with obesity and obesity-related diseases.

Objectives

The objectives of the study are to:

- evaluate the prognostic potential of the biomarkers in identifying subjects at risk for disease progression by correlating genetic predisposition, DNA methylation, protein and metabolite profiles with development of metabolic deterioration and other obesity-related complications.

- evaluate the capabilities of novel biomarkers at baseline in predicting treatment response to semaglutide by correlating protein and metabolite profiles at baseline with weight loss, body composition and other efficacy and safety parameters.

- evaluate the impact of genetic variation on obesity outcome, obesity-related complications and response to semaglutide therapy.

Methods and Study Design

The blood samples to be analysed in the present protocol have already been collected as bio-samples for future analyses in the completed clinical trials NN9536-4373 and NN9536-4374 sponsored by Novo Nordisk A/S. The samples are part of a research biobank owned and controlled by Novo Nordisk A/S and located at an external facility ([redacted]). The trials were randomised, double-blinded, global clinical trials and were completed and reported in Q2 2020 (see www.clinicaltrials.gov NCT03548935 and NCT03552757).

Trial NN9536-4373 was conducted at 129 sites in 16 countries as follows: Argentina, Belgium, Bulgaria, Canada, Denmark, Finland, France, Germany, India, Japan, Mexico, Poland, Russian Federation, Taiwan, United Kingdom, United States. This was a 68-week, randomised, double-blind, placebo-controlled, two-armed, parallel group, multi-centre, multinational clinical trial comparing semaglutide s.c. 2.4 mg once-weekly with semaglutide placebo once-weekly in subjects with overweight or obesity. Eligible subjects were randomised in a 2:1 manner to receive either semaglutide s.c 2.4 mg once-weekly or semaglutide placebo once-weekly as an adjunct to a reduced-calorie diet and increased physical activity. In total, 1961 patients were randomised in this trial, of which 1427 (941 semaglutide, 486 placebo) gave consent for the bio-banking of samples for future research.

Trial NN9536-4374 was conducted at 149 sites in 12 countries as follows: Argentina, Canada, Germany, Greece, India, Japan, Russian Federation, South Africa, Spain, United Arab Emirates, United Kingdom and United States. This was a 68-week, randomised, double-blinded, double dummy, placebo-controlled, multi-centre, multinational clinical trial in which eligible subjects were randomised in a 1:1:1 manner to receive either semaglutide s.c. 2.4 mg and semaglutide placebo II once-weekly; semaglutide s.c. 1.0 mg and semaglutide placebo I once-weekly; or semaglutide placebo I and semaglutide placebo II once-weekly; all as an adjunct to a reduced-calorie diet and increased physical activity. In total, 1210 patients were randomised in this trial, of which 694 (236 semaglutide 2.4 mg, 236 semaglutide 1.0 mg, 222 placebo) gave consent for the bio-banking of samples for future research.

The protocols for trials NN9536-4373 and NN9536-4376 are included in Appendix 1. The patient information and informed consent form for the clinical trial are included in Appendix 2 and the patient information and informed consent for the use of blood samples for future research is included in Appendix 3.

Bio-banked samples for future research were obtained at randomisation and at end of treatment at week 68.

The samples were processed and stored at -20°C at each investigational site prior to being transferred to [redacted] (stored at -80°C) located at [redacted]. All samples have been collected in an ethically compliant manner as part of Novo Nordisk-sponsored clinical trials, in compliance with the national law at each site of collection, and the samples have been lawfully transferred from each country to [redacted]. Samples will be subsequently transferred from [redacted] to the contracted vendors for analysis (see Page 7).

The sample material is considered appropriate for the request and appropriate for the intended use.

The serum samples will be analysed by:

- Proteomics - SomaLogic using the SomaScan Discovery platform (protein array) and by use of Mass Spectrometry

- Metabolomics - Metabolon's Precision Metabolomics™ LC-MS global metabolomics platform

The Human Peripheral Blood Mononuclear Cells in [redacted] tubes will be analysed by:

- Common genetic variation - [redacted], using the Illumina OmniExpress 24 Single Nucleotide Polymorphism (SNP) array

- Targeted gene sequencing of prespecified set of genes (please see below) - [redacted]

- DNA methylation: [redacted], using the Illumina Infinium EPIC Methylation assay (DNA methylation)

A total of 4,242 serum samples (1 mL volume per sample) will be sent to SomaLogic, Inc. (2945 Wilderness Place; Boulder, CO 80301, US) for proteomics analysis. The residual sample (approx. 0.5 mL) will then be transferred from SomaLogic, Inc. to Novo Nordisk A/S (Måløv, Denmark) for aliquoting and storage. A portion of each sample (approx. 0.25 mL) will then be shipped to the Novo Nordisk Foundation Center for Protein Research (University of Copenhagen, Denmark) for mass spectrometry analysis. Similar mass spectrometry analysis will be performed on the remaining samples (approx. 0.25 mL per sample) at Novo Nordisk A/S (Måløv). The mass spectrometry analysis is expected to take more than 7 days to complete; therefore a research biobank will be established at each location while this work is ongoing. The samples will be retained in the research biobank for no longer than 4 months after the completion of the analyses described in this protocol, i.e. the biobank will be closed no later than 31 Oct 2023. Any sample remaining after mass spectrometry has been completed will be destroyed, and documentation of sample destruction will be obtained from the Novo Nordisk Foundation Center for Protein Research. Transfer of personal data (including biosamples) to SomaLogic and to the Novo Nordisk Foundation Center for Protein Research will be in compliance with the Danish Data Protection Act (databeskyttelsesloven) and the General Data

Protection Regulation (GDPR; chapter V in particular) and only pseudonymised data will be transferred.

A total of 4,242 serum samples (1 mL volume per sample) will be sent to [redacted] for metabolomics analysis. Any sample remaining after analysis has been completed will be destroyed, and documentation of sample destruction will be obtained from [redacted].

A total of 1,704 DNA samples (1 mL volume per sample) will be sent to [redacted] for genetic analysis. The analysis is expected to take more than 7 days to complete; therefore a research biobank will be established at [redacted] while this work is ongoing. The samples will be retained in the research biobank for no longer than 4 months after the completion of the analyses described in this protocol, i.e. the biobank will be closed no later than 31 Oct 2023. Any sample remaining after analysis has been completed will be destroyed, and documentation of sample destruction will be obtained from [redacted].

Transfer of samples to [redacted] and [redacted] will be in compliance with the Danish Data Protection Act and the GDPR and only pseudonymised data will be transferred to the contracted laboratories.

SomaScan Discovery platform provided by SomaLogic, is a proteomic assay platform that is currently qualified for exploratory use in the clinic (research use only)[15]. The assay measures secreted proteins or peptides as well as unique protein or peptide markers which are released from each organ in the body under pathological or stressed conditions. The SomaScan® Assay uses Slow Off-Rate Modified Aptamers (called SOMAmer® reagents) to provide 7,000 highly reproducible measurements of circulating proteins from a single sample. These 7000 protein markers consist mostly of secreted proteins or peptides and unique protein or peptide markers which are released from each organ in the body under pathological or stressed conditions. The specific fingerprints of these protein or peptide markers are often associated with certain disease states.

Exploratory proteomic analysis using mass spectrometry will be performed in collaboration with researchers from the University of Copenhagen using a method that is currently qualified for exploratory use only. This method includes protocols for protein degradation and isolation and subsequent analysis by use of mass spectrometry[16]. A similar analysis will be performed at Novo Nordisk A/S (Måløv, Denmark) for validation purposes.

Precision Metabolomics™ LC-MS global metabolomics platform provided by [redacted], is a metabolomics platform using statistical approaches to assess the relative quantity and analysis of over 5,200 metabolites across 70 metabolic pathways. The samples are processed using ultra high-performance liquid chromatography/tandem accurate mass spectrometry (UHPLC/MS/MS) methods. These metabolites may help understand upstream gene expression and downstream function of metabolites in a particular biochemical pathway.

The Infinium OmniExpress-24 Kit analysed by [redacted] provides information on common genetic variation across 710,000 markers. This microarray has strategically selected markers needed to capture the greatest amount of common variation and does not assess rare variation

including variation influencing any genes on the American College of Medical Genetics and Genomics list of actionable variants[17]. The data originating from this array can assist understanding of the genetic interplay with response to semaglutide therapy and obesity-related complications.

Targeted Exome sequencing array analysed by [redacted] will be used to obtain information on targeted regions of the genome (<1% of the genome) that have previously been associated with obesity and cardiometabolic diseases.

Three methods have been used for identifying genes associated with cardiometabolic disease:

1. Genes previously found to be associated with severe early-onset obesity in children and adults were selected[18]. Both gene panels are in the public domain and used by academic and pharmaceutical researchers: https://nhsgms-panelapp.genomicsengland.co.uk/panels/130/v2.2 (Appendix 4a, n_{genes}=29) and https://uncoveringrareobesity.com/genepanel (Appendix 4b, n_{genes}=79).

2. Regions of the genome previously identified by genome-wide association studies (GWAS) For each cardiometabolic trait of interest (type 2 diabetes[19-23], body mass index[24], waist-hip ratio[25], trunk fat ratio[26], chronic kidney disease[27], heart failure[28], coronary artery disease[29], stroke[30], non-alcoholic steatohepatitis[31], osteoarthritis[32] and Alzheimer's disease[33]), we identified genetic variants associated at genome-wide significance (p-value $<5x10^{-8}$) from the most recent GWAS study. This p-value threshold provides a strict and internationally recognised threshold to elucidate significant genetic variants after accounting for the number of independent genetic segments across the genome[34,35]. Using this approach ensures that the genes targeted are from regions that are truly associated with cardiometabolic disease. In addition, the reference publications are from high-impact journals (*Nature Genetics*, *Nature Communications*, *Human Molecular Genetics*, *Circulation Research*) and authored by international leaders in the field of genetics of the respective cardiometabolic conditions.

   We used BED tools to identify genes located within 200kbp of the sentinel genetic variant (https://bedtools.readthedocs.io/en/latest/index.html) based on gene locations provided by Ensembl (http://feb2014.archive.ensembl.org/Homo_sapiens/Info/Index). Two additional steps were performed: first we restricted our gene list to protein-coding genes only, and second, we removed any genes that are on the ACMG list[17]. These approaches identified 7420 genes (Appendix 4c).

3. Genes selected based on previously published functional relationships with obesity and osteoarthritis. Firstly, hallmarks of obesity and osteoarthritis were defined. Hallmarks of disease have previously been described as an organising principle for rationalising the complexity of disease[36,37]. Hallmarks of obesity and osteoarthritis were defined as key biological pathways and processes underlying these diseases. Secondly, a curation process was carried out to identify scientific peer-reviewed articles that describe a link between a gene/protein and an obesity/osteoarthritis hallmark. This involved a prioritisation of articles e.g. for human relevance and a manual evaluation of the supporting data presented in each article. This approach led to selecting 185 articles used for identification of genes related to obesity and osteoarthritis. Further, an internal build knowledge graph combined data from the

literature mining with pathways and other publicly available databases (Ensembl[38], OpenTargets[39], PathwaysCommons[40], Uniprot[41]). Using this connectivity of genes, proteins, and diseases phenotypes within the graph, we additionally mined genes that functionally described associations to Obesity, Atherosclerosis, and Fatty Liver disease. In total, 912 genes were identified using the functional relationships with obesity and osteoarthritis (Appendix 4d).

Based on these approaches and due to overlap between genes identified by the 3 outlined methods, we have identified 7951 unique genes associated with obesity and cardiometabolic disease that we intend to analyse. We note that this is a limited fraction of the total number of genes in humans.

Whole exome sequencing will be used to derive genetic information from exonic regions and a filter will be implemented to restrict this mapping to the 7951 prespecified genes for our analyses. The sequenced reads will then be aligned and mapped to the reference genome. Once these sequence reads have been isolated, all other sequence reads will be destroyed, leaving no data outside of the prespecified 7951 genes. All further analyses will be performed on these genes in isolation.

Illumina Infinium EPIC Methylation assay provided by [redacted] is a methylation assay which provides quantitative array-based genome-wide methylation measurement at the single-CpG-site level. Genomic DNA will be extracted using automated and validated commercial methods and will be bisulfite converted using the EZ DNA methylation kit according to the manufacturer's protocol. This array allows methylation investigation of 850,000 CpG sites without the need for extensive DNA sequencing.

Name of analytical laboratories:

- Proteomics: SomaScan Discovery: SomaLogic, Inc. 2945 Wilderness Place; Boulder, CO 80301, US; Novo Nordisk Foundation Center for Protein Research, University of Copenhagen, Denmark; and Novo Nordisk A/S, Måløv, Denmark.
- Metabolomics: [redacted]
- Common genetic variation, targeted gene sequencing and DNA methylation: [redacted].

Statistical considerations

The table below provides the approximate number of stored samples available per treatment arm in the two clinical trials for use in the proposed studies for DNA methylation, genetics, proteomics and metabolomics. For DNA methylation a [redacted] sample from both baseline and end of trial will be analysed. For genetics (SNP array and targeted gene sequencing) one [redacted] sample from baseline or end of trial will be profiled. For proteomics and metabolomics, a baseline serum sample and a serum sample from week 68 (corresponding to the end of trial) for each individual will be profiled.

	**Trial ID: NN99536-4373**<br>BMI ≥30 kg/m^2 or ≥27 kg/m^2 and at least 1 comorbidity (excluding T2D)<br>(N=1950)			**Trial ID: NN99536-4374**<br>BMI ≥27 kg/m^2 and T2D<br>(N=1200)			
	Semaglutide 2.4 mg	Placebo	Total	Semaglutide 2.4 mg	Semaglutide 1.0 mg	Placebo	Total
**No of trial subjects with serum samples at randomisation and week 68**	941	486	**1427**	236	236	222	**694**
**No of trial subjects with [redacted] samples for DNA methylation analysis at baseline <u>and</u> week 68**	84	55	**139**	51	51	55	**157**
**No of trial subjects with [redacted] samples for genetic analysis at week 68**	594	303	**897**	160	178	173	**511**

Based on previous Omics biomarker studies, the samples sizes in the table above are deemed acceptable for high-dimensional data mining and investigating differential expression. These data may be combined with other datasets from previous and future Novo Nordisk studies.

Studying the effect of treatment on these biomarkers will be done using a Mixed Model for Repeated Measures (MMRM) either alone or in combination with the *limma* procedure. The prognostic potential of biomarkers will be evaluated using an area under the receiver operating

characteristic (AUROC) analysis. Predictive biomarkers will be investigated using linear models with an interaction term between biomarker and treatment. Adjustment for multiple testing across analyses will be done using a Bonferroni-adjustment.

In addition to studying single biomarkers, data mining will be performed among other using regularization (i.e., statistical learning) where prognostic/predictive model will be trained and internally validated from the data, and as such control the type I error rate.

Description of the donors

Trials NN9536-4373 and NN9536-4374 included adult subjects with overweight (BMI≥27 kg/m^2 and at least one comorbidity) or obesity (BMI≥30 kg/m^2) The full list of inclusion and exclusion criteria for the two trials is available in the protocols in Appendix 1.

Adverse effects, risks and inconvenience

The study will analyse human blood samples that were consented exclusively for future research and already collected in trials NN9536-4373 and NN9536-4374 and does not in any way interfere with the treatment or bear any risk or possibility of adverse effects for the study subjects. The total amount of blood drawn for this purpose of future research was less than 68 mL and the samples were taken while study subjects were already having blood samples taken as part of the clinical trial.

The results of the analyses proposed in the present project will not identify new health-related risks that could have any impact on treatment of the individual participant.

Information from patient records

The clinical data collected by Novo Nordisk as part of trial NN9536-4373 and NN9536-4374 are already available in-house at Novo Nordisk for this study. The clinical data includes age, sex, ethnicity and other demographic parameters as well as efficacy and safety data collected during the conduct of the trials. The subjects have already consented to collection of data from medical records as part of participation in the clinical trials NN9536-4373 and NN9536-4374. No other patient records (e.g., external hospital records) will be accessed as part of the present project.

Handling of personal data in the project

All information concerning research participants are protected in compliance with The Data Protection Act, the GDPR, and the Danish Health Act (Sundhedsloven).

Initiation of the project depends on approval from the Regional Committee on Health Research Ethics (Den Regionale Videnskabsetiske Komite).

Subject’s identity will remain confidential and the samples will be identified only by subject number, visit number and trial identification number. No direct identification of the subject is known to Novo Nordisk and therefore, only pseudonymised data will be processed.

Novo Nordisk is the data controller of all samples stored in the established biobank at [redacted], who acts as a data processor. Pseudonymised samples will be shipped from [redacted] to the contracted laboratories mentioned on page 5 and 8 for analysis (SomaLogic, Inc., United States; Novo Nordisk Foundation Center for Protein Research, Denmark; [redacted]). These laboratories thus act as data processors for Novo Nordisk. Furthermore, Novo Nordisk may provide clinical data collected as part of trials NN9536-4373 and NN9536-4374 to these analysis laboratories for the purpose of performing the contracted analyses and only pseudonymised data will be provided. Transfer of personal data (including bio-samples) to the contracted laboratories will be in compliance with the Data Protection Act and the GDPR (in particular chapter V for international transfers). Regarding the transfer of personal data (including bio-samples) specifically to SomaLogic, Inc. in the United States, the new Standard Contractual Clauses (SCCs) adopted by the European Commission (data controller for data processor) will be used as the transfer mechanism. Prior to the transfer, a Transfer Impact Assessment will be conducted, and supplementary measures (incl. pseudonymisation) implemented to protect the transferred personal data.

Economy, remuneration and other services

The study is initiated, sponsored, and conducted entirely by Novo Nordisk. Novo Nordisk also sponsored trials NN9536-4373 and NN9536-4374, from which the samples used in this study originates. No costs are payable to the patients for the use of their bio-samples and data in the present project.

The researchers at Novo Nordisk are employees of a company, which potentially has long-term economic interests in the described research project. Novo Nordisk will hold all rights for any patenting that may result from the data generated in the project. Novo Nordisk may use the data resulting from the project to generate new therapeutic drugs and thereby achieve economic profit.

Informed consent

Participants in trials NN9536-4373 and NN9536-4374 have already signed a separate informed consent agreeing to the use of blood samples for future research (Appendix 3). This consent specifies that the bio-samples will be used for investigation of: i) the study medicine; ii) obesity and related diseases; iii) genetics of obesity and other related diseases; and iv) new methods of testing. Moreover, the informed consent specifies that Novo Nordisk will not look at all genes (genetic code).

Novo Nordisk seeks an exemption for re-consent for the present research project based on the following considerations:

- The project does not involve health risks or impose strains on the research participants because the project uses only material from previous research projects (bio-samples that have already been collected as part of Novo Nordisk-sponsored clinical trials NN9536-4373 and NN9536-4374).

- The clinical trials NN9536-4373 and NN9536-4374 are already completed and were conducted in >3000 patients at multiple sites across several countries, meaning that disproportionate efforts would be required to obtain re-consent.

- There is a minimal risk of making new health findings that are clinically meaningful for individual patients.

Publication of study results

The results of the clinical trials under which the bio-samples for future use were collected have already been published (see www.clinicaltrials.gov NCT03548935 and NCT03552757). The proposed analyses on the bio-samples in the present project are exploratory. Results, whether positive, negative, or inconclusive, will be published in international peer-reviewed journals and/or as abstracts for oral or poster presentations at scientific conferences. Relevant results may also be made available to health authorities, including the European Medicines Agency and the US Food and Drug Administration, in connection with submission of drug approval applications. Any publication of patient demographics or clinical information will follow The Data Protection Act and the GDPR.

Ethical considerations

There is an unmet need for exploring novel prognostic and predictive biomarkers beyond BMI within obesity to better understand the disease and identify patients at highest risk of disease progression and in need of treatment.

As the study involves human bio-samples that already have been collected from participants in trials NN9536-4373 and NN9536-4374, the present project will not result in any risk or adverse effects for the patients. Moreover, by selecting a SNP array solely capturing common genetic variation with minor allele frequency >5% and sequencing only genetic loci previously associated with type 2 diabetes, body mass index, waist-hip ratio, trunk fat ratio, chronic kidney disease, heart failure, coronary artery disease, stroke, non-alcoholic steatohepatitis, osteoarthritis and Alzheimer’s disease, this protocol will adhere to the informed consent and does not include extensive or agnostic mapping of the genome.

Trials NN9536-4373 and NN9536-4374 were conducted in accordance with the Declaration of Helsinki (2013) and ICH Good Clinical Practice (2016).

The following documents have been appended:

Appendix 1: Study protocols for the Trials NN9536-4373 and NN9536-4374

Appendix 2: Participant Information and Informed Consent Form for the NN9536-4373 and NN9536-4374 Trials

Appendix 3: Participant Information and Informed Consent Form for the NN9536-4373 and NN9536-4374 Trials - samples for future research

Appendix 4a, 4b,4c,4c: List of genes to be sequenced using the three methods described in the text above.

References

1. Kelly T, Yang W, Chen CS, Reynolds K, He J. Global burden of obesity in 2005 and projections to 2030. Int J Obes (Lond) 2008;32(9):1431-7. DOI: 10.1038/ijo.2008.102.
2. Finkelstein EA, DiBonaventura M, Burgess SM, Hale BC. The costs of obesity in the workplace. J Occup Environ Med 2010;52(10):971-6. (In eng). DOI: 10.1097/JOM.0b013e3181f274d2.
3. Van Nuys K, Globe D, Ng-Mak D, Cheung H, Sullivan J, Goldman D. The association between employee obesity and employer costs: evidence from a panel of U.S. employers. Am J Health Promot 2014;28(5):277-85. (In eng). DOI: 10.4278/ajhp.120905-QUAN-428.
4. Guh DP, Zhang W, Bansback N, Amarsi Z, Birmingham CL, Anis AH. The incidence of co-morbidities related to obesity and overweight: a systematic review and meta-analysis. BMC Public Health 2009;9:88. (In eng). DOI: 10.1186/1471-2458-9-88.
5. Mechanick JI, Garber AJ, Handelsman Y, Garvey WT. American Association of Clinical Endocrinologists' position statement on obesity and obesity medicine. Endocr Pract 2012;18(5):642-8. DOI: 10.4158/EP12160.PS.
6. The American Society for Metabolic and Bariatric Surgery, The Obesity Society, The American Society of Bariatric Physicians and the American Association of Clinical Endocrinologists. Obesity is a Disease: Leading Obesity Groups Agree (Joint Press Release). 19 June (http://www.prnewswire.com/news-releases/obesity-is-a-disease-leading-obesity-groups-agree-212194851.html).
7. Rosenbaum M, Goldsmith R, Bloomfield D, et al. Low-dose leptin reverses skeletal muscle, autonomic, and neuroendocrine adaptations to maintenance of reduced weight. J Clin Invest 2005;115(12):3579-86. (In eng). DOI: 10.1172/JCI25977.
8. Hinkle W, Cordell M, Leibel R, Rosenbaum M, Hirsch J. Effects of reduced weight maintenance and leptin repletion on functional connectivity of the hypothalamus in obese humans. PLoS One 2013;8(3):e59114. (In eng). DOI: 10.1371/journal.pone.0059114.
9. Goldsmith R, Joanisse DR, Gallagher D, et al. Effects of experimental weight perturbation on skeletal muscle work efficiency, fuel utilization, and biochemistry in human subjects. Am J Physiol Regul Integr Comp Physiol 2010;298(1):R79-88. (In eng). DOI: 10.1152/ajpregu.00053.2009.
10. Sumithran P, Prendergast LA, Delbridge E, et al. Long-term persistence of hormonal adaptations to weight loss. N Engl J Med 2011;365(17):1597-604. DOI: 10.1056/NEJMoa1105816.
11. Lau J, Bloch P, Schäffer L, et al. Discovery of the once-weekly glucagon-like peptide-1 (GLP-1) analogue semaglutide. J Med Chem 2015;58(18):7370-80. (In eng). DOI: 10.1021/acs.jmedchem.5b00726.
12. Wilding JPH, Batterham RL, Calanna S, et al. Once-Weekly Semaglutide in Adults with Overweight or Obesity. N Engl J Med 2021;384(11):989. (In eng). DOI: 10.1056/NEJMoa2032183.
13. Davies M, Færch L, Jeppesen OK, et al. Semaglutide 2·4 mg once a week in adults with overweight or obesity, and type 2 diabetes (STEP 2): a randomised, double-blind, double-dummy, placebo-controlled, phase 3 trial. Lancet 2021;397(10278):971-984. (In eng). DOI: 10.1016/S0140-6736(21)00213-0.
14. Nimptsch K, Konigorski S, Pischon T. Diagnosis of obesity and use of obesity biomarkers in science and clinical medicine. Metabolism 2019;92:61-70. (In eng). DOI: 10.1016/j.metabol.2018.12.006.

15. Elskens JP, Elskens JM, Madder A. Chemical Modification of Aptamers for Increased Binding Affinity in Diagnostic Applications: Current Status and Future Prospects. Int J Mol Sci 2020;21(12). DOI: 10.3390/ijms21124522.
16. Mann M. The Origins of Organellar Mapping by Protein Correlation Profiling. Proteomics 2020;20(23):e1900330. DOI: 10.1002/pmic.201900330.
17. Miller DT, Lee K, Chung WK, et al. ACMG SF v3.0 list for reporting of secondary findings in clinical exome and genome sequencing: a policy statement of the American College of Medical Genetics and Genomics (ACMG). Genet Med 2021;23(8):1381-1390. DOI: 10.1038/s41436-021-01172-3.
18. Styne DM, Arslanian SA, Connor EL, et al. Pediatric Obesity-Assessment, Treatment, and Prevention: An Endocrine Society Clinical Practice Guideline. J Clin Endocrinol Metab 2017;102(3):709-757. DOI: 10.1210/jc.2016-2573.
19. Vujkovic M, Keaton JM, Lynch JA, et al. Discovery of 318 new risk loci for type 2 diabetes and related vascular outcomes among 1.4 million participants in a multi-ancestry meta-analysis. Nat Genet 2020;52(7):680-691. DOI: 10.1038/s41588-020-0637-y.
20. Mahajan A SNC, Zhang, W et al. Trans-ancestry genetic study of type 2 diabetes highlights the power of diverse populations for discovery and translation. 2020. DOI: 10.1101/2020.09.22.20198937.
21. Spracklen CN, Horikoshi M, Kim YJ, et al. Identification of type 2 diabetes loci in 433,540 East Asian individuals. Nature 2020;582(7811):240-245. DOI: 10.1038/s41586-020-2263-3.
22. Mahajan A, Taliun D, Thurner M, et al. Fine-mapping type 2 diabetes loci to single-variant resolution using high-density imputation and islet-specific epigenome maps. Nat Genet 2018;50(11):1505-1513. DOI: 10.1038/s41588-018-0241-6.
23. Mahajan A, Wessel J, Willems SM, et al. Refining the accuracy of validated target identification through coding variant fine-mapping in type 2 diabetes. Nat Genet 2018;50(4):559-571. DOI: 10.1038/s41588-018-0084-1.
24. Yengo L, Sidorenko J, Kemper KE, et al. Meta-analysis of genome-wide association studies for height and body mass index in approximately 700000 individuals of European ancestry. Hum Mol Genet 2018;27(20):3641-3649. DOI: 10.1093/hmg/ddy271.
25. Pulit SL, Stoneman C, Morris AP, et al. Meta-analysis of genome-wide association studies for body fat distribution in 694 649 individuals of European ancestry. Hum Mol Genet 2019;28(1):166-174. DOI: 10.1093/hmg/ddy327.
26. Rask-Andersen M, Karlsson T, Ek WE, Johansson A. Genome-wide association study of body fat distribution identifies adiposity loci and sex-specific genetic effects. Nat Commun 2019;10(1):339. DOI: 10.1038/s41467-018-08000-4.
27. Morris AP, Le TH, Wu H, et al. Trans-ethnic kidney function association study reveals putative causal genes and effects on kidney-specific disease aetiologies. Nat Commun 2019;10(1):29. DOI: 10.1038/s41467-018-07867-7.
28. Shah S, Henry A, Roselli C, et al. Genome-wide association and Mendelian randomisation analysis provide insights into the pathogenesis of heart failure. Nat Commun 2020;11(1):163. DOI: 10.1038/s41467-019-13690-5.
29. van der Harst P, Verweij N. Identification of 64 Novel Genetic Loci Provides an Expanded View on the Genetic Architecture of Coronary Artery Disease. Circ Res 2018;122(3):433-443. DOI: 10.1161/CIRCRESAHA.117.312086.
30. Malik R, Chauhan G, Traylor M, et al. Multiancestry genome-wide association study of 520,000 subjects identifies 32 loci associated with stroke and stroke subtypes. Nat Genet 2018;50(4):524-537. DOI: 10.1038/s41588-018-0058-3.

31. Anstee QM, Darlay R, Cockell S, et al. Genome-wide association study of non-alcoholic fatty liver and steatohepatitis in a histologically characterised cohort(). J Hepatol 2020;73(3):505-515. DOI: 10.1016/j.jhep.2020.04.003.
32. Boer CG, Hatzikotoulas K, Southam L, et al. Deciphering osteoarthritis genetics across 826,690 individuals from 9 populations. Cell 2021;184(18):4784-4818 e17. DOI: 10.1016/j.cell.2021.07.038.
33. Wightman DP, Jansen IE, Savage JE, et al. A genome-wide association study with 1,126,563 individuals identifies new risk loci for Alzheimer's disease. Nat Genet 2021;53(9):1276-1282. DOI: 10.1038/s41588-021-00921-z.
34. Pe'er I, Yelensky R, Altshuler D, Daly MJ. Estimation of the multiple testing burden for genomewide association studies of nearly all common variants. Genet Epidemiol 2008;32(4):381-5. DOI: 10.1002/gepi.20303.
35. Fadista J, Manning AK, Florez JC, Groop L. The (in)famous GWAS P-value threshold revisited and updated for low-frequency variants. Eur J Hum Genet 2016;24(8):1202-5. DOI: 10.1038/ejhg.2015.269.
36. Hanahan D, Weinberg RA. Hallmarks of cancer: the next generation. Cell 2011;144(5):646-74. DOI: 10.1016/j.cell.2011.02.013.
37. (https://www.cellsignal.com/science-resources/hallmarks-of-ndg).
38. Howe KL, Achuthan P, Allen J, et al. Ensembl 2021. Nucleic Acids Res 2021;49(D1):D884-D891. DOI: 10.1093/nar/gkaa942.
39. Ochoa D, Hercules A, Carmona M, et al. Open Targets Platform: supporting systematic drug-target identification and prioritisation. Nucleic Acids Res 2021;49(D1):D1302-D1310. DOI: 10.1093/nar/gkaa1027.
40. Rodchenkov I, Babur O, Luna A, et al. Pathway Commons 2019 Update: integration, analysis and exploration of pathway data. Nucleic Acids Res 2020;48(D1):D489-D497. DOI: 10.1093/nar/gkz946.
41. UniProt C. UniProt: the universal protein knowledgebase in 2021. Nucleic Acids Res 2021;49(D1):D480-D489. DOI: 10.1093/nar/gkaa1100.
