# Supplementary material for: Proteomic changes upon treatment with semaglutide in individuals with obesity
Source: Nat Med. 2025 Jan 3;31(1):267–77. doi: 10.1038/s41591-024-03355-2 (PMC11750704; doi:10.1038/s41591-024-03355-2)
Supplement: Supplementary file 2 — Reporting Summary [file 41591_2024_3355_MOESM2_ESM.pdf]

nature portfolio

Corresponding author(s): Abd A. Tahrani

Last updated by author(s): Sept 26, 2024

Reporting Summary

Nature Portfolio wishes to improve the reproducibility of the work that we publish. This form provides structure for consistency and transparency in reporting. For further information on Nature Portfolio policies, see our Editorial Policies and the Editorial Policy Checklist.

Statistics

For all statistical analyses, confirm that the following items are present in the figure legend, table legend, main text, or Methods section.

n/a	Confirmed	
☐	☒	The exact sample size (*n*) for each experimental group/condition, given as a discrete number and unit of measurement
☐	☒	A statement on whether measurements were taken from distinct samples or whether the same sample was measured repeatedly
☐	☒	The statistical test(s) used AND whether they are one- or two-sided<br>*Only common tests should be described solely by name; describe more complex techniques in the Methods section.*
☐	☒	A description of all covariates tested
☐	☒	A description of any assumptions or corrections, such as tests of normality and adjustment for multiple comparisons
☐	☒	A full description of the statistical parameters including central tendency (e.g. means) or other basic estimates (e.g. regression coefficient) AND variation (e.g. standard deviation) or associated estimates of uncertainty (e.g. confidence intervals)
☐	☒	For null hypothesis testing, the test statistic (e.g. *F*, *t*, *r*) with confidence intervals, effect sizes, degrees of freedom and *P* value noted<br>*Give P values as exact values whenever suitable.*
☒	☐	For Bayesian analysis, information on the choice of priors and Markov chain Monte Carlo settings
☒	☐	For hierarchical and complex designs, identification of the appropriate level for tests and full reporting of outcomes
☐	☒	Estimates of effect sizes (e.g. Cohen's *d*, Pearson's *r*), indicating how they were calculated

Our web collection on statistics for biologists contains articles on many of the points above.

Software and code

Policy information about availability of computer code

Data collection	No software was used for data collection
Data analysis	Data were processed in R using the SomaDataIO package for loading raw proteomics data from .adat files and tidyverse packages for data processing. For the comparison with observational cohort data, this was performed using cameraPR (a "pre-ranked" version of the competitive gene set method camera), which is part of the Bioconductor limma package. CameraPR was run with default parameters using limma v3.52.4. R v4.3.1; SomaDataIO v6.0.0; tidyverse v2.0.0; Limma v3.52.4. All code is available at: DOI: 10.5281/zenodo.13356055

For manuscripts utilizing custom algorithms or software that are central to the research but not yet described in published literature, software must be made available to editors and reviewers. We strongly encourage code deposition in a community repository (e.g. GitHub). See the Nature Portfolio guidelines for submitting code & software for further information.

Data

Policy information about availability of data

All manuscripts must include a data availability statement. This statement should provide the following information, where applicable:

- Accession codes, unique identifiers, or web links for publicly available datasets
- A description of any restrictions on data availability
- For clinical datasets or third party data, please ensure that the statement adheres to our policy

Proteomic results and summary association data are available through a dashboard available here: https://step-proteomics.azurewebsites.net/ as well as in

Supplementary Tables. Individual participant data from the STEP 1 and STEP 2 clinical trials can be shared in datasets in a de-identified and anonymized format. Access request proposals can be found at https://www.novonordisk-trials.com/. deCODE: Ferkingstad, E., et al. Large-scale integration of the plasma proteome with genetics and disease. Nat. Genet. 53, 1712–1721 (2021). The data used in the gene set enrichment analysis are available in Supplementary Table 11 in Ferkingstad et al. GWAS summary statistics for aptamers are available at https://www.decode.com/summarydata/. Hallmark: Liberzon, A., et al. The Molecular Signatures Database (MSigDB) hallmark gene set collection. Cell Syst. 1, 417–425 (2015). Data are available at https://www.gsea-msigdb.org/gsea/msigdb.

Human research participants

Policy information about studies involving human research participants and Sex and Gender in Research.

Reporting on sex and gender

Sex was self-reported in the STEP 1 and STEP 2 trials.

Population characteristics

Adult men and women randomized into the STEP 1 (with overweight/obesity without T2D) and STEP 2 (with overweight/obesity and T2D) trials. Please see Table 1 in the manuscript. Of the 1,311 participants from STEP 1, 72.8% (n=955) were female. Of the 645 participants from STEP 2, 49.8% (n=321) were female. Most participants were white, with a mean age of 47.5 in STEP 1 and 56.3 in STEP 2. Mean body weight was 106.0 kg in STEP 1, and 100.0 kg in STEP 2. Mean BMI was 37.9 kg/m2 in STEP 1 and 35.7 kg/m2 in STEP 2. In STEP 1, 43.5% of participants had prediabetes vs none in STEP 2. Baseline characteristics of participants in this study were similar to those of the overall study populations of the published STEP 1 (Wilding, J.P.H., et al. Once-weekly semaglutide in adults with overweight or obesity. N. Engl. J. Med. 384, 989–1002 (2021)) and STEP 2 trial (Davies, M., et al. Semaglutide 2.4 mg once a week in adults with overweight or obesity, and type 2 diabetes (STEP 2): a randomised, double-blind, double-dummy, placebo-controlled, phase 3 trial. Lancet 397, 971–984 (2021)).

Recruitment

This analysis involved data from participants who gave consent for the collection of biosamples, with serum samples available at baseline and at week 68. STEP 1 was conducted across 129 sites in 16 countries in Asia, Europe, North America, and South America, as described in a previous publication (Wilding, J.P.H., et al. Once-weekly semaglutide in adults with overweight or obesity. N. Engl. J. Med. 384, 989–1002 (2021)). STEP 2 participants were recruited from 149 outpatient clinics in 12 countries across Europe, North America, South America, the Middle East, South Africa, and Asia, as described in Davies, M., et al. Semaglutide 2.4 mg once a week in adults with overweight or obesity, and type 2 diabetes (STEP 2): a randomised, double-blind, double-dummy, placebo-controlled, phase 3 trial. Lancet 397, 971–984 (2021). In the current analysis, we utilized fasting serum samples collected at baseline and end of treatment in a large subset of participants from STEP 1 and STEP 2 to investigate the effects of semaglutide treatment on the circulating proteome.

Ethics oversight

The study protocol for the proteomic analyses was approved by the ethics committee for the Region of Southern Denmark (no. H-21046833).

Note that full information on the approval of the study protocol must also be provided in the manuscript.

Field-specific reporting

Please select the one below that is the best fit for your research. If you are not sure, read the appropriate sections before making your selection.

☒ Life sciences ☐ Behavioural & social sciences ☐ Ecological, evolutionary & environmental sciences

For a reference copy of the document with all sections, see nature.com/documents/nr-reporting-summary-flat.pdf

Life sciences study design

All studies must disclose on these points even when the disclosure is negative.

Sample size

A total of 3,171 male and female participants were included in the STEP 1 and STEP 2 trials. Of these, 1,956 participants (STEP 1, n = 1,311; STEP 2, n = 645) consented to aptamer-based proteomic analyses using the SomaScan® assay v4.1 (SomaLogic, Boulder, CO, USA). After filtering for sample availability at both timepoints, individuals not on treatment at study end in both arms, and vendor quality control, 1,728 participants (STEP 1, n = 1,133; STEP 2, n = 595) remained. For the majority of the analyses, only the placebo and semaglutide 2.4 mg arms from the STEP 2 trial were analysed (n = 395). Baseline characteristics of consented participants are shown in Table 1 and were similar to those of the overall study populations of the STEP 1 and STEP 2 trials. Sample sizes were estimated for the clinical endpoints, please refer to the STEP 1 and STEP 2 publications for further details on this. No further power calculations were performed in connection with the proteomics study.

Data exclusions

Subjects with samples missing (at baseline or at week 68) or not on treatment at week 68 were excluded. Furthermore, samples that failed QC by SomaLogic were excluded.

Replication

Data from two clinical trials (STEP 1 and STEP 2) were used to understand and compare the effects of semaglutide on the circulating proteome. To further corroborate our findings, clinical trial proteomic data were integrated with proteomic results from observational cohorts including deCODE. No explicit replication of the presented findings was performed. In general, a conservative approach was taken (e.g. family-wise error control using Bonferroni-Holm) and internal consistency was observed for many findings across individual aptamers (e.g. see gene set enrichment analyses).

Randomization

Samples for the analysis were derived from two phase 3 randomized controlled trials, STEP 1 and STEP 2, in which participants were randomized to either semaglutide or placebo. Individuals were randomized to receive either placebo or semaglutide (1.0 mg or 2.4 mg).

Please refer to the original STEP publications for further details regarding randomization.

Blinding	Both STEP 1 and STEP 2 trials were double blinded. Please refer to the original STEP publications for further details regarding blinding. In our analysis, we employed SomaLogic's normalization procedure, including the adaptive normalization by maximum likelihood step, which was used for the SomaScan® data set for all analyses as recommend by SomaLogic. Data were processed in R using the SomaDataIO package for loading raw proteomics data from .adat files and tidyverse packages for data processing. After filtering on samples passing SomaLogic quality control (RowCheck==TRUE) and selecting aptamers targeting human proteins, the remaining data covering 7,289 aptamers were log10 transformed and, for each aptamer, all measurements standardized using the mean and standard deviation (SD) of samples obtained at baseline.

Reporting for specific materials, systems and methods

We require information from authors about some types of materials, experimental systems and methods used in many studies. Here, indicate whether each material, system or method listed is relevant to your study. If you are not sure if a list item applies to your research, read the appropriate section before selecting a response.

Materials & experimental systems

n/a	Involved in the study
☒	☐ Antibodies
☒	☐ Eukaryotic cell lines
☒	☐ Palaeontology and archaeology
☒	☐ Animals and other organisms
☐	☒ Clinical data
☒	☐ Dual use research of concern

Methods

n/a	Involved in the study
☒	☐ ChIP-seq
☒	☐ Flow cytometry
☒	☐ MRI-based neuroimaging

Clinical data

Policy information about clinical studies

All manuscripts should comply with the ICMJE guidelines for publication of clinical research and a completed CONSORT checklist must be included with all submissions.

Clinical trial registration	STEP 1, NCT03548935; STEP 2, NCT03552757
Study protocol	STEP 1 - see Wilding et al. N Engl J Med 2021;384:989-1002; STEP 2 - see Davies et al. Lancet. 2021;397(10278):971-984
Data collection	Participants who gave consent for the collection of biosamples, had serum samples collected at baseline and week 68. All biosamples were taken from overnight-fasted (>8 hours) participants.
Outcomes	The SomaScan® assay v4.1 (Soma Logic, Boulder, CO, USA) was used for profiling of ~6,400 unique human proteins encompassing a diverse set of biological processes (e.g., cancer, inflammation, and cardiovascular function) and secreted, intracellular, and extracellular proteins/domains (e.g., receptors, kinases, growth factors, and hormones).
